# An attempt to synthesize a terthienyl-based analog of indacenedithiophene (IDT): unexpected synthesis of a naphtho[2,3-*b*]thiophene derivative†

**DOI:** 10.1039/d1ra00659b

**Published:** 2021-03-08

**Authors:** Cătălin C. Anghel, Ioan Stroia, Alexandra Pop, Atilla Bende, Ion Grosu, Niculina D. Hădade, Jean Roncali

**Affiliations:** Babeș-Bolyai University, Faculty of Chemistry and Chemical Engineering, Department of Chemistry, SOOMCC 11 Arany Janos str. 400028 Cluj-Napoca Romania niculina.hadade@ubbcluj.ro; University of Bucharest, Faculty of Chemistry, Department of Organic Chemistry, Biochemistry and Catalysis, Research Centre of Applied Organic Chemistry 90-92 Panduri Street RO-050663 Bucharest Romania; National Institute for Research and Development of Isotopic and Molecular Technologies 67-103 Donath str. Cluj-Napoca RO-400293 Romania; Group Linear Conjugated Systems, Moltech Anjou CNRS, University of Angers 2Bd lavoisier 49045 Angers France jeanroncali@gmail.com

## Abstract

We report herein our attempt to synthesize an analog of indacenedithiophene (IDT) based on a tetraphenylhexyl substituted, covalently bridged *syn*-terthienyl unit. Instead of the expected compound the adopted synthetic route led to the formation of an unexpected, new naphtho[2,3-*b*]thiophene derivative. The structure of this compound was fully characterized by NMR and HRMS as well as single crystal X-ray diffraction and its electronic properties have been analyzed by UV-vis absorption spectroscopy and cyclic voltammetry. A possible mechanism for the formation of this compound is also proposed on the basis of detailed theoretical investigations.

## Abstract 2

Serendipitous synthesis of a novel naphtho[2,3-*b*]thiophene derivative. Detailed investigation of the structure and properties as well as a possible mechanism for its formation.

## Graphical abstract

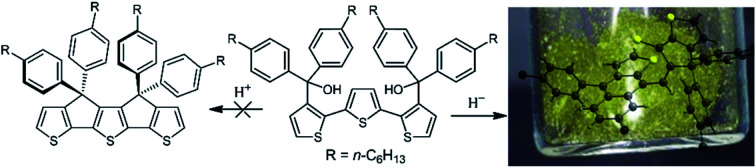

## Introduction

The design of molecular functional π-conjugated systems in view of their application as active materials in organic (opto)electronic devices such as organic field-effect transistors (OFETs), organic light-emitting diodes (OLEDs) and organic photovoltaics (OPV) has been a focus of intense research effort in the last few decades.^1–6^^.^ These various applications require a precise control of the electronic properties of the materials at molecular, mesoscopic and macroscopic levels including the energy levels of the frontier orbitals and their difference, light absorption and emission as well as electron and/or hole mobility. Among the various synthetic principles for the control of the energy gap of polyaromatic π-conjugated systems, the covalent rigidification of the carbon backbone has been shown to be highly effective due to the simultaneous planarization of the conjugated structure associated, in some cases, with a reduction of bond length alternation^7–12^ In the case of thiophene-based π-conjugated systems, this approach has been successfully applied to various classes of systems including poly(bithiophenes)thienylene polymers and oligomers and push–pull NLO-phores.^8,11–13^

**Fig. 1:**
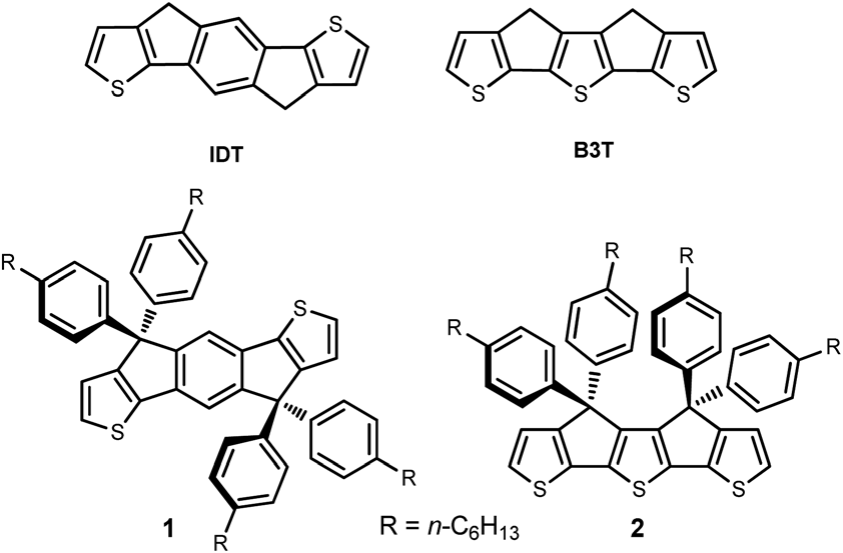
Structure of the indacenodithiophene (IDT) and covalently bridged terthienyl (B3T) skeleton (top) as well as the structure of IDT-based NFA 1 and target B3T-based compound 2.

During the past few years, the strong emergence of non-fullerene electron acceptor materials (NFAs) has generated impressive progress in the efficiency of OPV cells.^14–16^ Intensive research effort in synthetic chemistry has shown that the most efficient NFAs are built around rigid and planar fused ring systems such as indacenodithiophene (IDT) (Fig. 1) which represents the archetype of this kind of building blocks.^17,18^ It has been shown already that covalent bridging of terthienyl (3T) into a fully planar *syn* conformation (B3T, Fig. 1) yields to a considerable reduction of the HOMO–LUMO gap, compared to the free analog, due among other factors to a complete elimination of rotational disorder.^19^ Based on the structural analogy of IDT and B3T we have undertaken the synthesis of compound 2 (Fig. 1, *i.e.* the B3T analog of the tetra-phenylhexyl substituted IDT1), which is the building block generally used for practical syntheses due to its higher solubility.^15–18^ It was anticipated that the lower resonance energy of thiophene *vs.* benzene could lead to a new class of NFAs with red-shifted absorption. Such IR absorbing acceptors materials are of high potential interest in the context of transparent OPV cells now actively investigated for possible applications in building integrated photovoltaics (BIPV).^20–23^

**Scheme 1:**
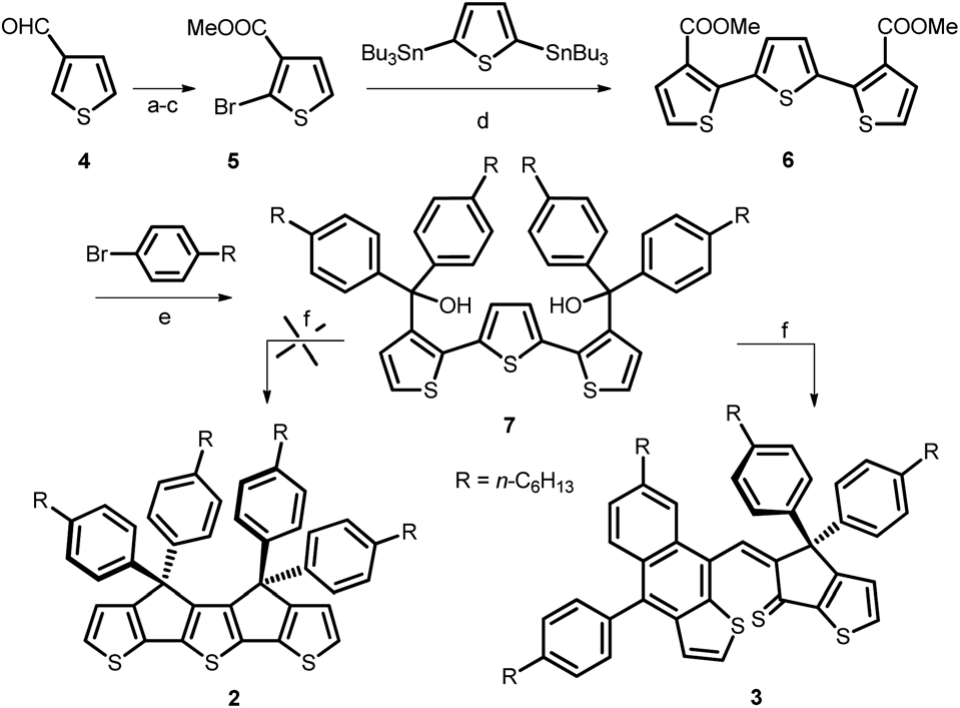
Tentative synthetic route for the preparation of the target compound 2. Reagents and conditions: (a) KOH, AgNO_3_, 70 °C, 1 h, 84%; (b) *n*-BuLi (2.2 eq.) −78 °C, 20 min, Br_2_, (1.1 eq.), −78 °C to r.t., overnight, 55%; (c) SOCl_2_, MeOH, reflux, 3 days, 80%; (d) Pd(PPh_3_)_4_, toluene, reflux, 2 days, 62%; (e) Mg, dry THF, reflux, 24 h, 70%. (f) *p*-Toluenesulfonic acid (PTSA), DCM, rt, 12 h, 10%.

We report herein an attempt to synthesize compound 2. Although, this attempt remained unsuccessful, the adopted synthetic approach led to the formation of an unexpected, new naphtho[2,3-*b*]thiophene derivative 3 (Scheme 1). Noteworthy, thiophene-functionalized acenes are known to have good optical and electronic properties and also found applications as active materials in opto-electronic devices.^24,25^ However, to date, there is a limited number of synthetic methods available to build naphtho[2,3-*b*]thiophene skeleton that usually involve multi-step synthesis.^26,27^ Therefore, we considered of interest to investigate the structure and properties of this new derivative.

## Results and discussion

In a previous report, unsubstituted B3T was obtained by coupling the enolate of a cyclopenta[*b*]thiophene followed by ring closure with Lawesson reagent.^19^ However, this method has no practical use to obtain the phenylhexyl-substituted derivative 2 needed for further synthetic work. Consequently, we adapted the procedure already reported for the synthesis of ladder poly(*p*-phenylene) and IDT^9,10,17^ based on the cyclization of the terthiophene-based diol 7 as key intermediate (Scheme 1). This compound was obtained in five steps starting from 3-formyl-thiophene 4. The terthiophene skeleton was built up using a double Stille reaction between 2,5-bis(tri-*n*-butylstannyl)thiophene and bromoester 5 prepared from 4, by oxidation, bromination and esterification. Treatment of the terthiophene diester 6 with the Grignard reagent obtained *in situ* from *p*-bromohexylbenzene and Mg, afforded 7 in good yield. Unexpectedly, Brønsted acid-catalyzed cyclization of 7 did not resulted in the formation of target molecule 2, but led to the naphtho[2,3-*b*]thiophene compound 3 in 10% yield. A similar result was obtained when the cyclization reaction was performed in presence of BF_3_ as catalyst.

**Scheme 2:**
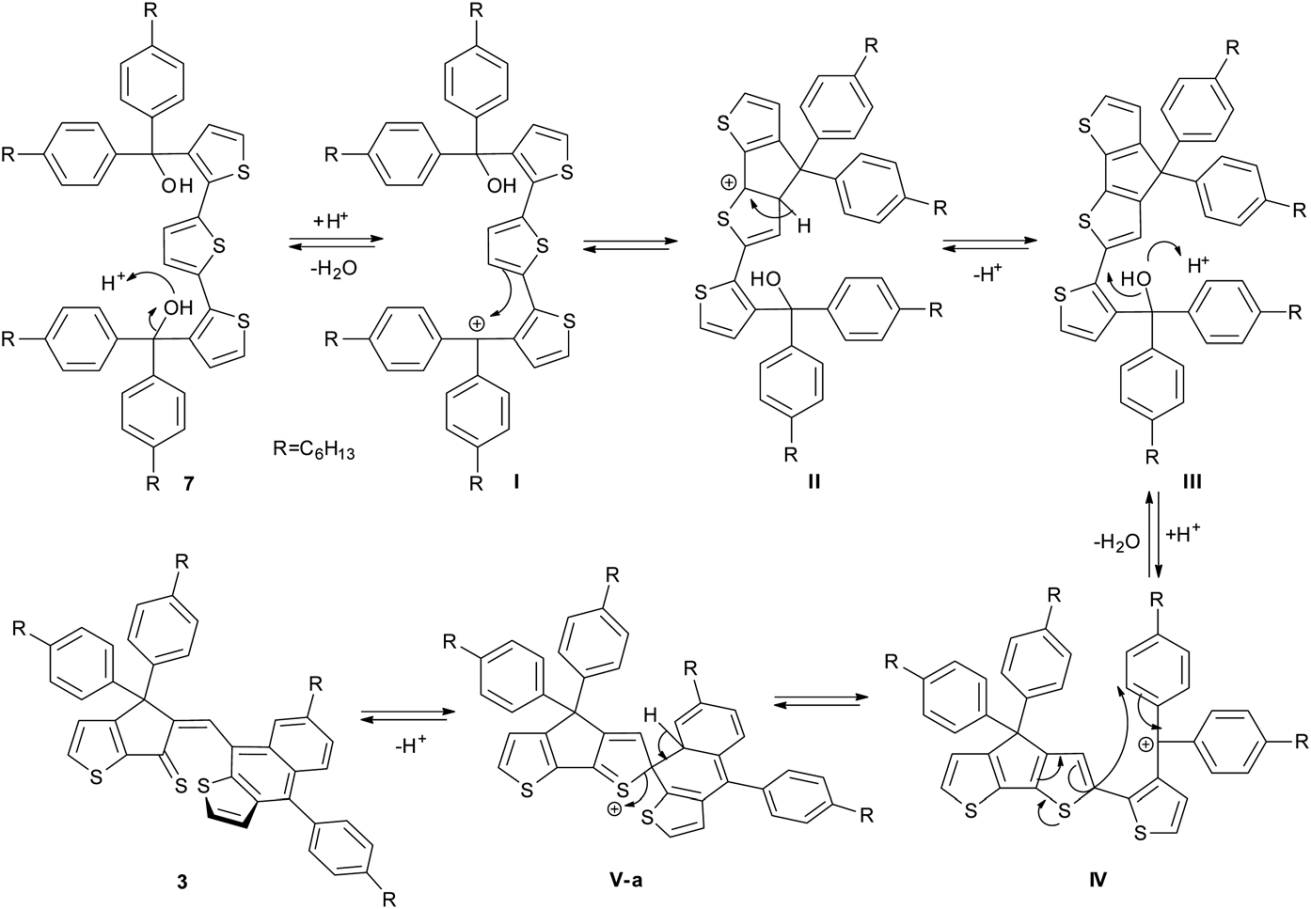
Proposed mechanism for the formation of 3.

The structure of 3 was clearly identified from ^1^H-, ^13^C- and 2D-NMR spectra (Fig. 3 and ESI†) as well as HRMS and single crystal X-ray diffraction. The formation of compound 3 could be explained through the mechanism proposed in Scheme 2: protonation of the tertiary alcohol, elimination of water followed by electrophilic aromatic substitution of the thiophene proton from the closest position to obtain intermediate III. Next, we propose formation of the tertiary, benzyl carbocation IV-a, similar to cation I formed in the first step of the mechanism. However, most probably because of the steric hindrance, instead of the expected electrophilic aromatic substitution of the thiophene ring, the attack does not occur directly onto the carbocation but in the *ortho* position of one of the phenyl groups (transformation IV-a → V-a). Finally, elimination of the proton afforded compound 3.

In order to gain more insights into the mechanism of formation of compound 3 and to explain the outcome of the cyclization reaction (see Scheme S1 in ESI† for the proposed mechanism for the formation of compound 2), a DFT computational study of the electrophilic aromatic substitution in IV (Scheme 2) has been performed. Since the mechanism involves several reaction steps, our theoretical investigation limits to the description of the IV → V steps, where the structure III already lost the hydroxyl group. Accordingly, the total charge of the systems IV and V was set +1*e*.

**Fig. 2:**
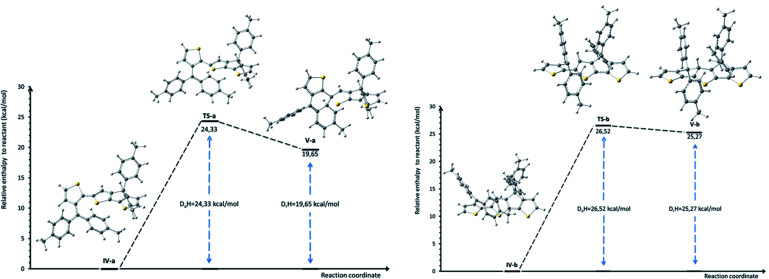
Calculated relative enthalpies profile (298.15 K; 1 atm) for the proposed electrophilic aromatic substitution step in intermediates IV-a and IV-b yielding to 3 (left) and 2 (right). Absolute and relative enthalpies of the intermediates and transition states are given in ESI, (Table S1†).

Theoretical calculations results seem to confirm the experimental findings indicating that both activation and reaction enthalpy favor the formation of 3*vs.*2. Thus, intermediate IV was found to display two minima, IV-a (local minimum) and IV-b (global minimum) with only 1.33 kcal mol^−1^ enthalpy difference (Fig. 2 and Table S1 in ESI.†). Starting from IV-a or IV-b the reaction mechanism can follow two different pathways for the electrophilic substitution step yielding to V-a or V-b, intermediates in the obtaining of 3 and 2 respectively, with activation enthalpy of 24.33 kcal mol^−1^ and 26.52 kcal mol^−1^ respectively (Fig. 2). Moreover, calculated global enthalpies for the transformation 7 to 3 and 7 to 2 (see Table S2†), are also in agreement with experimental results, indicating 16.33 lower conformational energy for 3 than 2.

**Fig. 3:**
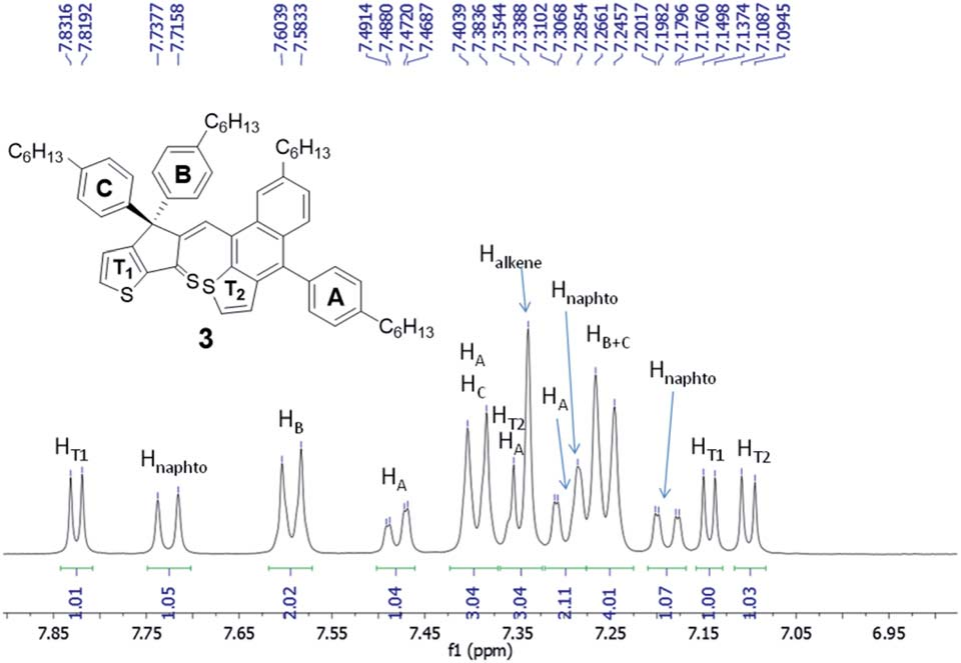
Fragment of ^1^H-NMR spectrum of compound 3 (DCM-*d*_2_, 600 MHz, r.t.).

Careful analysis of 1D and 2D NMR spectra of 3 was in agreement with the formation of a single diastereoisomer. The double bond was found to be in *Z* configuration as inferred by ROESY-NMR (*i.e.* correlation between the signal corresponding to H_alkene_ at 7.34 ppm and aromatic protons of ring B at 7.59 ppm, see also Fig. S20†). In addition, ^1^H-NMR spectrum of 3 (Fig. 3 and S16†), recorded in CD_2_Cl_2_, at room temperature, correspond to a rigid structure where the rotation of naphtho[2,3-*b*]thienyl unit and phenylene ring A is hindered as proved by the heterotopicity of the phenylene groups B and C (signals at 7.59, 7.39 and 7.25 ppm) as well as the diastereotopicity of the protons corresponding to phenylene ring A that give 4 signals at 7.48, 7.39, 7.34 and 7.30 ppm. These data suggest that compound 3 is obtained as a mixture of enantiomers (*i.e.* atropisomers) formed as result of the hindered rotation of naphtho[2,3-*b*]thienyl unit. Moreover, the diasteropicity of the protons belonging to ring A is in agreement with a hindered rotation of this unit too. Indeed, the calculated energy for the rotation around the single bond that connect aromatic ring A and naphtho[2,3-*b*]thienyl unit was found 17.33 kcal mol^−1^ (see Fig. S3 in ESI†)

**Fig. 4:**
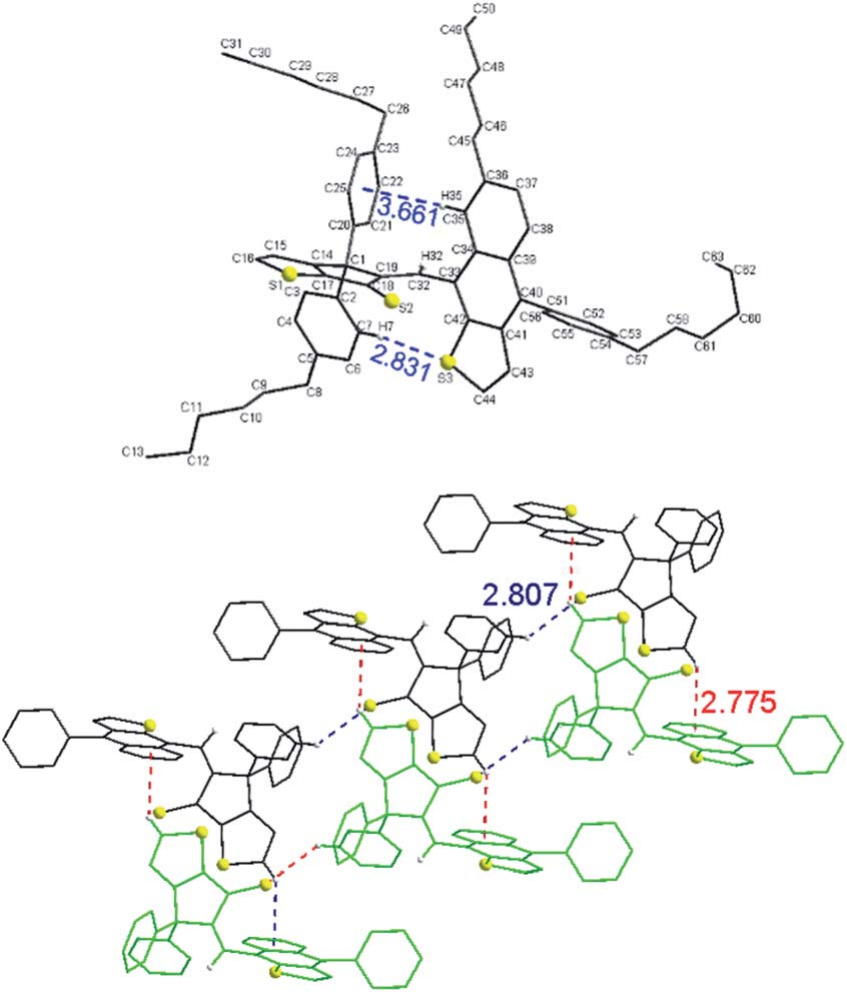
Single-crystal molecular structure of compound 3 (top). Fragment of a double-layer chain (red dotted lines – interchain interactions; blue dotted lines – intrachain interactions) view along *b* crystallographic axis (bottom). Hydrogen atoms are omitted for clarity.

Crystals of 3 suitable for single-crystal X-ray diffraction were obtained by slow evaporation from a mixture of DCM and MeCN. The diastereoselective formation of *Z* isomer of 3 was also confirmed in solid state by single crystal X-ray diffraction (Fig. 4, top). The structure is stabilized by intramolecular edge-to-face interaction between naphtho[2,3-*b*]thienyl unit and a phenyl ring (*i.e.* distance from C–H(35) to centroid of phenyl ring is 3.66 Å and the angle between naphtho[2,3-*b*]thienyl and phenyl units about 75°) and C–H⋯S interaction (*i.e.* distance C–H(7)–S(3) of 2.83 Å).

Examination of the crystal lattice revealed several interesting features. The enantiomers of 3 forms, by enantioselective recognition, infinite chains through C–H <svg xmlns="http://www.w3.org/2000/svg" version="1.0" width="37.000000pt" height="16.000000pt" viewBox="0 0 37.000000 16.000000" preserveAspectRatio="xMidYMid meet"><metadata> Created by potrace 1.16, written by Peter Selinger 2001-2019 </metadata><g transform="translate(1.000000,15.000000) scale(0.014583,-0.014583)" fill="currentColor" stroke="none"><path d="M80 440 l0 -40 320 0 320 0 0 40 0 40 -320 0 -320 0 0 -40z M880 440 l0 -40 320 0 320 0 0 40 0 40 -320 0 -320 0 0 -40z M1680 440 l0 -40 320 0 320 0 0 40 0 40 -320 0 -320 0 0 -40z"/></g></svg> S=C interactions (*i.e.* distance C–H(4) to S(2) is 2.81 Å) as well as hydrophobic interactions between hexyl groups. Two such chains containing the opposite enantiomers are connected through C–Hπ interactions (distance C–H(16) to centroid 2.77 Å) (Fig. 4, bottom). 3D packing of the crystals (Fig. S1†) displayed double-layer chains of opposite enantiomers connected by hydrophobic interactions between hexyl groups.

The optical and electrochemical properties of compound 3 were investigated by absorption and emission spectroscopy as well as cyclic voltammetry. The absorption properties of 3 were determined in dichloromethane solution. The UV-vis absorption spectrum of 3 displays three bands at 273 nm, 370 nm and 516 nm with molecular extinction coefficients of 4.06 × 10^4^, 2.25 × 10^4^ and 4.71 × 10^3^, respectively. Surprisingly, compound 3 does not show any significant emission bands when excited with the absorption maxima wavelength.

**Fig. 5:**
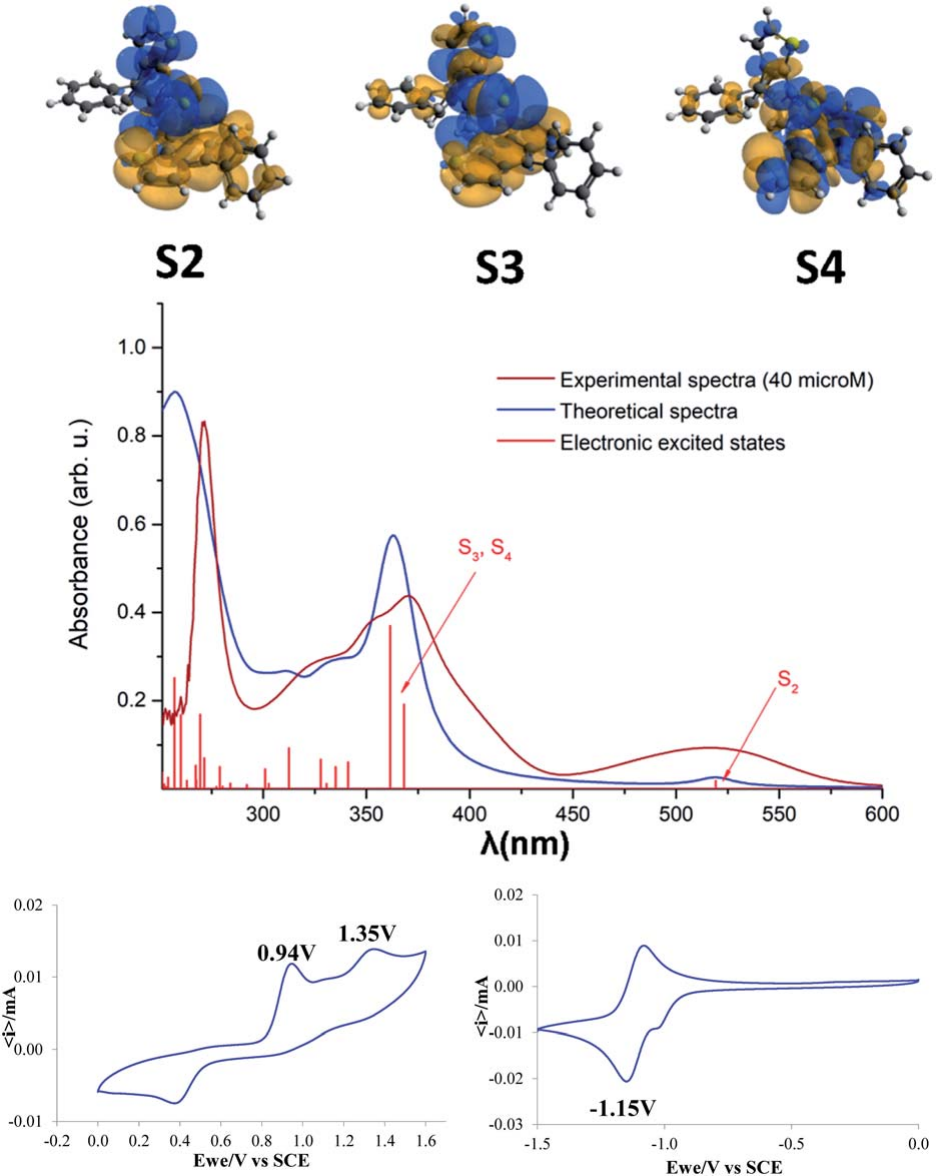
Natural difference orbitals (NDOs) for the S_2_, S_3_ and S_4_ electronic states (top). Experimental (40 μM, DCM) and theoretical UV-vis absorption spectra as well as the electronic excited states of compound 3 (middle, *i.e.* for better comparison with the measured UV-vis absorption spectra, a scaling factor of 1.12 was used for the energy value of each electronic excited state). Cyclic voltammograms of 3 (10^−3^ M) recorded at room temperature in 0.10 M *n*-Bu_4_NPF_6_, acetonitrile/DCM (8 : 2, v/v), Pt electrodes, scan rate = 100 mV s^−1^. All potentials were reported relative to a saturated calomel electrode (SCE).

Theoretical calculations were also performed in order to assign the observed absorption bands. The experimental and theoretical UV-vis absorption spectra, as well as the electronic excited states are presented in Fig. 5. Theoretical calculations have revealed that the first, relatively broad band from the experimental spectrum, at 519 nm is given by the second electronic excited state (S_2_). One should mention that the first excited state (S_1_ = 684 nm) has a very small oscillator strength, thus its peak is missing from the spectrum. The second band from the experimental spectrum around 370 nm is the contribution of the third and fourth electronic excited states with absorption wavelengths of 368 nm and 361 nm. The spectral range of 300–350 nm includes eight further excited states, but their oscillator strengths are weaker so they give only a shoulder to this band. The third, narrow but intense band in the experimental spectrum around 273 nm is the contribution of several higher level electronic excited states. In order to get more details about the nature of the electronic excited states the natural difference orbitals based on the difference density between ground and excited states was also computed for the S_2_, S_3_ and S_4_ electronic states (Fig. 5, top). Analyzing the orbitals shapes, one can conclude that S_2_ covers the so-called charge-transfer excitation from the naphtho[2,3-*b*]thienyl unit to the thiophene and C=S fragments, S_3_ is a mixture of local and charge-transfer excitations effects where mainly the naphtho[2,3-*b*]thienyl unit and the C=S fragment are involved, while S_4_ shows only a local excitation character. From the charge separation point of view S_2_ and S_3_ show the most proper behavior as they allow efficient charge separation induced by the photon absorption.

The electrochemical properties of 3 were analyzed in acetonitrile/DCM = 8/2 (v/v) as the solvent, using 0.10 M tetra-*n*-butylammoniumhexafluorophosphate as the supporting electrolyte (Fig. 5, bottom). The CV traces indicate two successive oxidation processes with anodic peak potentials at 0.94 and 1.35 V tentatively assigned to the cation radical and di-cation of the phenyl-naphtothiophene block respectively. The cathodic wave observed at 0.40 V in the reverse scan suggests the reduction of a product of degradation of the highest oxidized state. The negative potential region displays a reversible reduction process corresponding to the formation of a radical-anion with a cathodic peak potential at −1.15 V. The energy levels of the frontier orbitals of compound 5 (*E*_HOMO_ = −5.85 eV and *E*_LUMO_ = −3.94 eV) were calculated from the onset of oxidation and reduction potential, respectively.

## Conclusions

In summary our attempt to synthesize a rigid terthienyl-based analog of IDT leads to the unexpected formation of a new naphtho[2,3-*b*]thiophene compound. This derivative is most probably formed as result of the steric hindrance in the cyclization step an assumption supported by theoretical calculation. The structure of the new compound was clearly determined by NMR, single-crystal X-ray diffraction and high-resolution mass-spectrometry. The results are in agreement with the diastereoselective formation of *Z* isomer, as pair of enantiomers, resulting from hindered rotation of naphtho[2,3-*b*]thienyl unit, in both solid state and solution. Noteworthy, these enantiomers show enantioselective recognition in solid state. Work is currently under progress in order to synthesize target molecule by alternative routes as well as other similar structures based on different central units such as rigid dithienothiophenes.

## Experimental

### General experimental data

Commercially available reagents were used without further purification. Thin layer chromatography (TLC) was performed on silica gel 60 coated aluminium F_254_ plates and visualised by UV irradiation at 254 nm or by staining with 2,4-dinitrophenylhydrazine solution. Preparative column chromatography was carried out using silica gel 60 (0.040–0.063 mm) from Merck. The NMR spectra were recorded on Bruker Avance 400 MHz or Bruker Avance 600 MHz spectrometers. Chemical shifts (*δ*) are reported in parts per million (ppm) using residual solvent peak as internal reference. High resolution mass spectra were recorded on a Thermo Scientific (LTQ XL, Orbitrap) spectrometer, in positive ion mode, using Electrospray or APCI techniques. DFT computational study of the electrophilic aromatic substitution in IV (Scheme 2) has been performed using Gaussian 09 package,^28^ employing the dispersion corrected form of B3LYP exchange-correlation functional (B3LYP-D3, with D3 standing for Grimme's dispersion corrections)^29^ and the valence triple-zeta Def2-TZVP basis set.^30,31^ All geometries of interest were fully optimized, in dichloromethane as solvent (using default SCRF method) without any symmetry constraints. Moreover, vibrational frequencies analysis was performed in order to characterize the nature of the stationary points (*i.e.* minima and transition state), and for the calculation of zero-point energy corrections and thermal corrections. According to the vibrational analysis, all intermediate geometries correspond to minima and all transition states have only one imaginary frequency. The ultrafine integration grid was also used for all calculations. In addition, using B3LYP-D3/Def2-SVP method,^29^ we performed IRC calculations in order to demonstrate that each transition state connects the reactant and the product that are involved in electrophilic aromatic substitution mechanism (see Fig. S4†).

In order to calculate the theoretical UV-vis absorption spectrum of 3, the electronic excited states were computed in the framework of density functional theory considering the M06-2X^32^ exchange-correlation functional and the def2-TZVP^33^ basis set as is implemented in the Orca^34,35^ software package. The molecular graphics were created using the Avogadro^36^ molecular editor and visualizer software. The first 30 excited states were calculated, considering the dichloromethane as the solvent environment. For better comparison with the measured UV-vis absorption spectra, a scaling factor of 1.12 was used for the energy value of each electronic excited state. ESI crystallographic data for this paper, CCDC 2045572.†

### General procedure for synthesis of methyl 2-bromothiophene-3-carboxylate (5)

#### (a) Thiophene-3-carboxylic acid^37^

In a round bottom flask NaOH (3.5 g, 88 mmol, 3.5 eq.) is dissolved in water (15 mL). AgNO_3_ (7.5 g, 44 mmol, 1.75 eq.) dissolved in water (15 mL) is added, when the brown Ag_2_O is formed. 3-Thiophenecarboxaldehyde (2 mL, 25 mmol, 1 eq.) is added and the mixture is stirred for 1 hour. The Ag_2_O is filtered and is washed with water (15 mL). The filtrate is concentrated until its' volume is halved, and is acidified (pH = 1), when a white solid precipitate. The mixture is placed in a refrigerator for 12 hours and after that it is filtered. The white solid is washed with water and dried (2 g, 68%). ^1^H-NMR (400 MHz, MeOH-*d*_4_) *δ* (ppm): 8.19 (dd, 1H, ^3^*J* = 3.0 Hz, ^4^*J* = 1.2 Hz, H_Ar_), 7.49 (dd, 1H, ^3^*J* = 5.0 Hz, ^4^*J* = 1.2 Hz, H_Ar_), 7.45 (dd, 1H, ^3^*J* = 5.0 Hz, ^4^*J* = 3.0 Hz, H_Ar_). ^13^C-APT-NMR (100 MHz, MeOH-*d*_4_) *δ* (ppm): 166.1, 135.5, 134.1, 128.9, 127.4.

#### (b) 2-Bromothiophene-3-carboxylic acid^38^

In a two-neck round bottom, under argon, 3-thiophencarboxylic acid (0.5 g, 3.9 mmol, 1 eq.) is dissolved in dry THF (20 mL). The mixture is cooled down to −78 °C, *n*-BuLi (4.9 mL, 8.6 mmol, 2.2 eq.) is added dropwise during a period of 20 min. After 45 min Br_2_ (0.22 mL, 4.3 mmol, 1.1 eq.) is added dropwise, the mixture is further kept at −78 °C for 1 hour and then allowed to warm to r.t. overnight. After that the reaction mixture is acidified (pH = 1) and the compound is extracted with AcOEt (3 × 20 mL). The reunited organic phases are washed with brine, dried on MgSO_4_ and the solvent is evaporated. The compound is purified by recrystallization from MeOH : H_2_O = 4 : 1, when a white-yellowish solid (0.44 g, 55%) is obtained. ^1^H-APT-NMR (400 MHz, MeOH-*d*_4_)*δ* (ppm): 7.43 (d, 1H, ^3^*J* = 5.8 Hz, H_Ar_), 7.36 (d, 1H, ^3^*J* = 5.8 Hz, H_Ar_). ^13^C-NMR (100 MHz, MeOH-*d*_4_) *δ* (ppm): 164.9, 133.1, 130.6, 127.6, 120.4.

#### (c) Methyl 2-bromothiophene-3-carboxylate, adapted protocol^39^

In a round bottom flask 2-bromo-3-thiophencarboxylic acid (0.4 g, 1.92 mmol, 1 eq.) is dissolved in MeOH (40 mL). Thionyl chloride (0.85 mL, 11.52 mmol, 6 eq.) is added dropwise, and the mixture is refluxed until completion. After that it is cooled down and water (40 mL) is added. The compound is extracted with ethyl ether (4 × 20 mL). The reunited organic phases are washed with NaHCO_3_ saturated solution, brine and dried on MgSO_4_, and the solvent evaporated. The compound is purified by column chromatography (silica, AcOEt : petroleum ether = 1 : 8, *R*_f_ = 0.62), when a brownish liquid (0.34 g, 80%) is obtained. ^1^H-NMR (600 MHz, CDCl_3_) *δ* (ppm): 7.36 (d, 1H, ^3^*J* = 5.8 Hz, H_Ar_), 7.22 (d, 1H, ^3^*J* = 5.8 Hz, H_Ar_), 3.88 (s, 3H, C*H*_3_). ^13^C-APT-NMR (150 MHz, CDCl_3_) *δ* (ppm): 162.6, 131.1, 129.5, 126.0, 120.1, 52.1.

### General procedure for synthesis of dimethyl [2,2′:5′,2′′-terthiophene]-3,3′′-dicarboxylate (6)

In a two-neck round bottom flask, under argon, 2,5-bis(tributylstannyl)thiophene (0.58 mL, 1.05 mmol, 1 eq.) is dissolved in dry toluene (40 mL). Methyl 2-bromothiophene-3-carboxylate (5) (0.7 g, 3.15 mmol, 3 eq.) is added and the mixture is purged for 30 min with argon. The catalyst Pd(PPh_3_)_4_ (0.23 g, 0.2 mmol, 20%) is added, the mixture is further purged for 15 min and is refluxed for 2 days under argon. After cooling, a NaF saturated solution (40 mL) is added and the mixture is stirred for 2 hours. The compound is extracted with AcOEt (3 × 20 mL). The reunited organic phases are filtered on celite, washed with brine, dried on MgSO_4_ and the solvent is evaporated. The compound is purified by column chromatography (silica, AcOEt : petroleum ether = 1 : 8, *R*_f_ = 0.32), when an orange crystalline solid is obtained (0.23 g, 62%).

Mp = 90–92 °C,

^1^H-NMR (600 MHz, CDCl_3_) *δ* (ppm): 7.49 (d, 2H, ^3^*J* = 5.4 Hz, H_Ar_), 7.41 (s, 2H, H_Ar_), 7.21 (d, 1H, ^3^*J* = 5.4 Hz, H_Ar_), 3.85 (s, 3H, OC*H*_3_).

^13^C-APT-NMR (150 MHz, CDCl_3_) *δ* (ppm): 163.6, 142.9, 136.2, 130.7, 129.3, 127.7, 124.3, 51.9.

### General procedure for synthesis of [2,2′:5′,2′′-terthiophene]-3,3′′-diylbis(bis(4-hexylphenyl)methanol) (7)

In a two-neck flask equipped with a condenser, under argon, magnesium turnings (0.34 g, 15.4 mmol, 6.6 eq.) are suspended in dry THF (50 mL). The magnesium is activated with 2–3 drops of 1,2-dibromoethane. After that 1-bromo-4-hexylbenzene (3.4 g, 14 mmol, 6 eq.) dissolved in dry THF (20 mL) is added dropwise, during a period of 30 minutes, generating the Grignard reagent *in situ*. Dimethyl [2,2′:5′,2′′-terthiophene]-3,3′′-dicarboxylate (6) (0.85 g, 2.33 mmol, 1 eq.) dissolved in dry THF (20 mL) is added dropwise and the mixture is refluxed for 24 hours. The mixture is then cooled down and is quenched with a NH_4_Cl saturated solution (100 mL). The compound is extracted with AcOEt (3 × 100 mL). The reunited organic phases are washed with brine, dried on MgSO_4_ and the solvent is evaporated. The compound is purified by column chromatography (silica, AcOEt : petroleum ether = 1 : 8, *R*_f_ = 0.42), when an orange viscous oil (1.7 g, 70%) is obtained.

^1^H-NMR (600 MHz, CD_2_Cl_2_) *δ* (ppm): 7.11 (overlapped signals, 18H, H_Ar_), 6.43 (d, 2H, ^3^*J* = 5.4 Hz, H_Ar_), 6.33 (s, 2H, H_Ar_) 3.11 (s, 2H, O*H*), 2,59 (t, 8H, ^3^*J* = 7.7 Hz, C*H*_2_), 1.59 (m, 8H, C*H*_2_), 1.34–1.27 (overlapped signals, 24H, C*H*_2_), 0.87 (t, 12H, ^3^*J* = 6.8 Hz, C*H*_3_).

^13^C-APT-NMR (150 MHz, CD_2_Cl_2_) *δ* (ppm): 146.3, 145.2, 142.8, 137.5, 132.0, 131.9, 129.0, 128.4, 127.9, 124.2, 80.7, 36.1, 32.3, 32.0, 29.6, 23.2, 14.4.

ESI(+)-HRMS (*m*/*z*): calculated for C_62_H_76_O_2_S_3_: 971.4900, found 971.4952 [M + Na^+^]; 1920.9941, found 1921.0020 [2M + Na^+^].

### General procedure for synthesis of (*Z*)-5-((7-hexyl-4-(4-hexylphenyl)naphtho[2,3-*b*]thiophen-9-yl)methylene)-4,4-bis(4-hexylphenyl)-4*H*-cyclopenta[*b*]thiophene-6(5*H*)-thione (3)

In a round bottom flask [2,2′:5′,2′′-terthiophene]-3,3′′-diyl-bis(bis(4-hexylphenyl)methanol) (7) (0.83 g, 0.875 mmol, 1 eq.) is dissolved in DCM (30 mL). Para-toluene sulfonic acid (0.33 g, 1.75 mmol, 2 eq.) is added and the mixture is stirred at room temperature for 12 h. The mixture is filtered to separate the PTSA, and the solvent is evaporated. The compound is purified by column chromatography (silica, DCM : petroleum ether = 1 : 4, *R*_f_ = 0.44; DCM : petroleum ether : Et_3_N = 1 : 4 : 0.05 for the packing of the column), followed by slow crystallization from DCM/EtOH at low temperature, leading to green-yellow needles (80 mg, 10%).

Mp = 154–156 °C, *R*_f_ = 0.44 (DCM : petroleum ether = 1 : 4).

^1^H-NMR (600 MHz, CD_2_Cl_2_) *δ* (ppm): 7.82 (d, 1H, ^3^*J* = 5.0 Hz, H_Ar_), 7.72 (d, 1H, ^3^*J* = 8.8 Hz, H_Ar_), 7.59 (d, 2H, ^3^*J* = 8.3 Hz, H_Ar_), 7.48 (dd, 1H, ^3^*J* = 7.8 Hz, ^4^*J* = 1.7 Hz, H_Ar_), 7.39 (overlapped signals, 3H, H_Ar_), 7.35–7.33 (overlapped signals, 3H, H_Ar_), 7.31–7.28 (overlapped signals, 2H, H_Ar_), 7.25 (d, 4H, ^3^*J* = 7.2 Hz, H_Ar_), 7.19 (dd, 1H, ^3^*J* = 8.8 Hz, ^4^*J* = 1.7 Hz, H_Ar_), 7.14 (d, 1H, ^3^*J* = 5.0 Hz, H_Ar_), 7.10 (d, 1H, ^3^*J* = 5.7 Hz, H_Ar_), 2.74 (t, 2H, ^3^*J* = 7.8 Hz, C*H*_2_), 2.67–2.62 (overlapped signals, 4H, C*H*_2_), 2.59 (t, 2H, ^3^*J* = 7.6 Hz, C*H*_2_), 1.73 (m, 2H, C*H*_2_), 1.67–1.60 (overlapped signals, 4H, C*H*_2_), 1.57 (m, 2H, C*H*_2_), 1.44 (m, 2H, C*H*_2_), 1.40–1.28 (overlapped signals, 22H, C*H*_2_), 0.92 (t, 3H, ^3^*J* = 7.0 Hz, C*H*_3_), 0.90–0.85 (overlapped signals, 9H, C*H*_3_). ^13^C-APT-NMR (150 MHz, CD_2_Cl_2_) *δ* (ppm): 213.3, 165.8, 158.0, 157.9, 143.03, 143.00, 142.8, 142.7, 141.7, 140.7, 139.6, 138.3, 137.2, 136.5, 135.5, 135.2, 131.3, 131.2, 129.8, 129.22, 129.16, 129.15, 128.9, 128.8, 128.5, 127.4, 127.3, 127.1, 125.7, 124.7, 124.5, 123.3, 64.9, 36.7, 36.4, 36.2, 36.1, 32.4, 32.32, 32.31, 32.1, 32.0, 31.6, 29.8, 29.70, 29.65, 29.6, 23.3, 23.24, 23.21, 23.19, 14.5, 14.4. APCI(+)-HRMS (*m*/*z*): calculated for C_62_H_72_S_3_: 913.4869, found 913.4832 [M + H^+^].

## Author contributions

Cătălin C. Anghel – synthesis and compound characterization, Ioan Stroia and Atilla Bende – theoretical calculation, Alexandra Pop – X-ray analysis, Ion Grosu, Niculina D. Hădade and Jean Roncali – conceptualization, supervision, writing.

## Conflicts of interest

There are no conflicts to declare.

## Supplementary Material

RA-011-D1RA00659B-s001

RA-011-D1RA00659B-s002
