# Supplementary material for: An attempt to synthesize a terthienyl-based analog of indacenedithiophene (IDT): unexpected synthesis of a naphtho[2,3-b]thiophene derivative
Source: RSC Adv. 2021 Mar 8;11(17):9894–900. doi: 10.1039/d1ra00659b (PMC8695407; doi:10.1039/d1ra00659b)
Supplement: RA-011-D1RA00659B-s001 [file RA-011-D1RA00659B-s001.pdf]

Electronic Supplementary Material (ESI) for RSC Advances.
This journal is © The Royal Society of Chemistry 2021

Supporting Information

for

An attempt to synthesize a terthienyl-based analog of indacenedithiophene (IDT): unexpected synthesis of a naphtho[2,3-*b*]thiophene derivative.

by

Cătălin C. Anghel,[a,b] Ioan Stroia,[a] Alexandra Pop,[a] Atilla Bende,[c] Ion Grosu,[a] Niculina D. Hădade,[a,*] Jean Roncali[a,d,*]

[a]Babeș-Bolyai University, Faculty of Chemistry and Chemical Engineering, Department of Chemistry and SOOMCC, Cluj-Napoca, 11 Arany Janos str., 400028, Cluj-Napoca, Romania, e-mail: niculina.hadade@ubbcluj.ro (N. Hădade);
[b]University of Bucharest, Faculty of Chemistry; Department of Organic Chemistry, Biochemistry and Catalysis, Research Centre of Applied Organic Chemistry, 90-92 Panduri Street, RO-050663 Bucharest, Romania;
[c]National Institute for Research and Development of Isotopic and Molecular Technologies, 67-103 Donath str., RO-400293, Cluj-Napoca, Romania;
[c]Group Linear Conjugated Systems, Moltech Anjou CNRS, University of Angers, 2Bd lavoisier,49045 Angers, France, e-mail: jeanroncali@gmail.com (J. Roncali)

Summary

Scheme S1. Proposed mechanism for the formation of the expected compound 2 4

Figure S1. Crystal packing of compound 3 – view along axis a 4

Figure S2. UV-VIS spectra of compound 3 on spun-cast film on ITO from a DCM solution .. 5

Figure S3. Calculated relative enthalpies profile for the rotation of the phenyl group attached to the naphtho[2,3-*b*]thiophene unit in compound 3 5

Table S1. Calculated enthalpies for the intermediates involved in the formation of compounds 2 and 3 6

Table S2. Calculated enthalpies for compounds involved in global transformations 7 to 3 and 7 to 2, respectively 6

Figure S4. IRC plot for the path a (TS-a connects IV-a and V-a minima) (top) and path b (TS-b connects IV-b and V-b minima, bottom). The IRC calculations were performed at B3LYP-D3/Def2-SVP level of theory, in dichloromethane as solvent, without any symmetry constraints. 7

Figure S5. ^{1}H NMR (MeOH-d_4, 400 MHz) spectrum of thiophene-3-carboxylic acid 8

Figure S6. ^{13}C-APT-NMR (MeOH-d_4, 100 MHz) spectrum of thiophene-3-carboxylic acid... 8

Figure S7. ^{1}H NMR (MeOH-d_4, 400 MHz) spectrum of 2-bromothiophene-3-carboxylic acid9

Figure S8. ^{13}C-APT-NMR (MeOH-d_4, 100 MHz) spectrum of 2-bromothiophene-3-carboxylic acid 9
Figure S9. ^{1}H NMR ($CDCl_3$, 600 MHz) spectrum of compound 5 10
Figure S10. ^{13}C-APT NMR ($CDCl_3$, 150 MHz) spectrum of compound 5 10
Figure S11. ^{1}H NMR ($CDCl_3$, 600 MHz) spectrum of compound 6 11
Figure S12. ^{13}C-APT NMR ($CDCl_3$, 150 MHz) spectrum of compound 6 11
Figure S13. ^{1}H NMR (CD_2Cl_2, 600 MHz) spectrum of compound 7 12
Figure S14. ^{13}C-APT NMR (CD_2Cl_2, 150 MHz) spectrum of compound 7 12
Figure S15. ESI(+)-HRMS spectrum of 7 13
Figure S16. ^{1}H NMR(CD_2Cl_2, 600 MHz) spectrum of compound 3 13
Figure S17. ^{13}C APT (CD_2Cl_2, 150 MHz) spectrum of compound 3 14
Figure S18. APCI(+)-HRMS spectrum of 3 14
Figure S19. COSY (H,H)-NMR (CD_2Cl_2, 600 MHz) spectrum of compound 3: aromatic region 15
Figure S20. ROESY NMR (CD_2Cl_2, 600 MHz) spectrum of compound 3: aromatic region.. 15
Figure S21. HSQC NMR (CD_2Cl_2, 600 MHz) spectrum of compound 3: aromatic region 16
Figure S22. HMBC NMR (CD_2Cl_2, 600 MHz) spectrum of compound 3: aromatic region ... 16
Crystal structure determination 17
Table S3. Crystal data and structure refinement for 3 18
Figure S23. DFT calculated structure of TS-rot 19
Figure S24. DFT calculated structure of compound 7 19
Figure S25. DFT calculated structure of intermediate III 20
Figure S26. DFT calculated structure of intermediate IV-a 20
Figure S27. DFT calculated structure of TS-a 21
Figure S28. DFT calculated structure of intermediate V-a 21
Figure S29. DFT calculated structure of compound 3 22
Figure S30. DFT calculated structure of intermediate IV-b 22
Figure S31. DFT calculated structure of TS-b 23
Figure S32. DFT calculated structure of intermediate V-b 23
Figure S33. DFT calculated structure of compound 2 24
Table S4. DFT coordinates of TS-rot 24
Table S5. DFT coordinates of compound 7 26
Table S6. DFT coordinates of intermediate III 28
Table S7. DFT coordinates of intermediate IV-a 30

Table S8. DFT coordinates of TS-a 32
Table S9. DFT coordinates of intermediate V-a 34
Table S10. DFT coordinates of compound 3 36
Table S11. DFT coordinates of intermediate IV-b 38
Table S12. DFT coordinates of TS-b 40
Table S13. DFT coordinates of intermediate V-b 42
Table S14. DFT coordinates of compound 2 45
Table S15. DFT coordinates of water 47

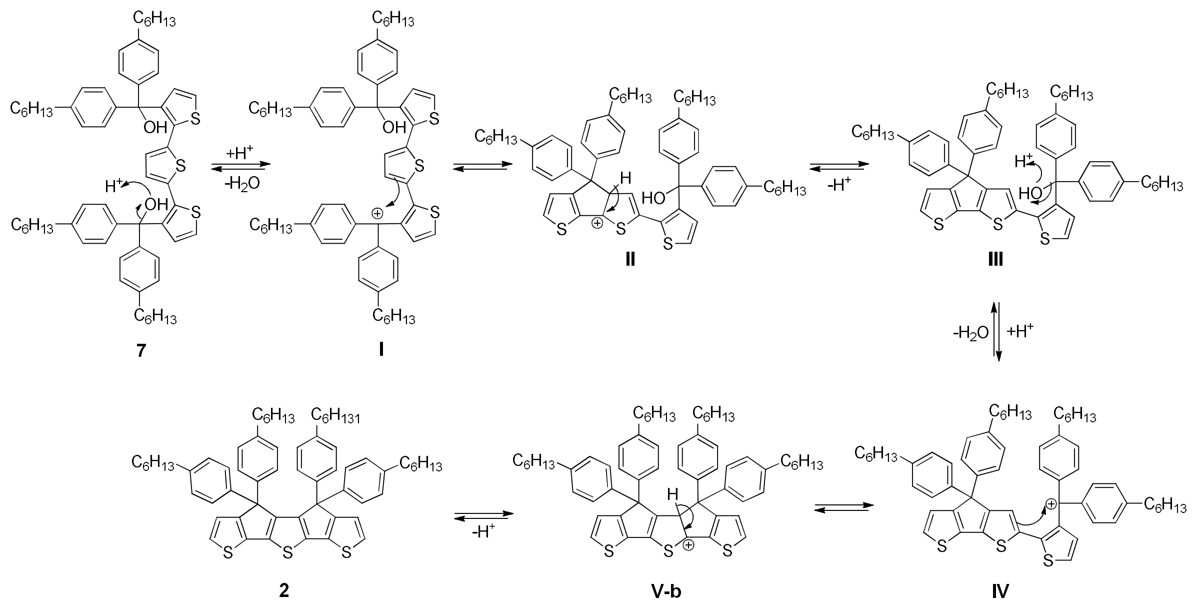

Scheme S1. Proposed mechanism for the formation of the expected compound 2

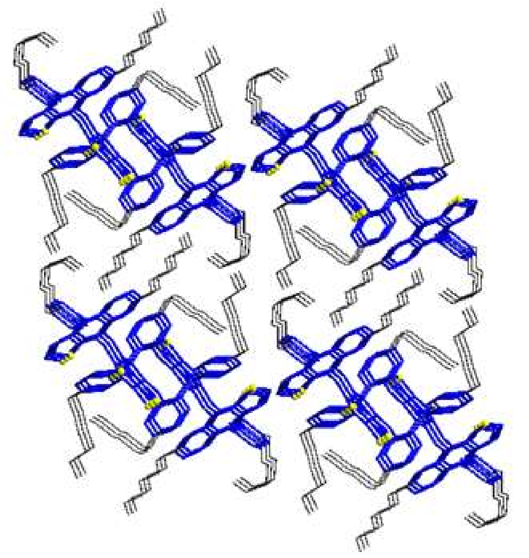

Figure S1. Crystal packing of compound 3 – view along axis a

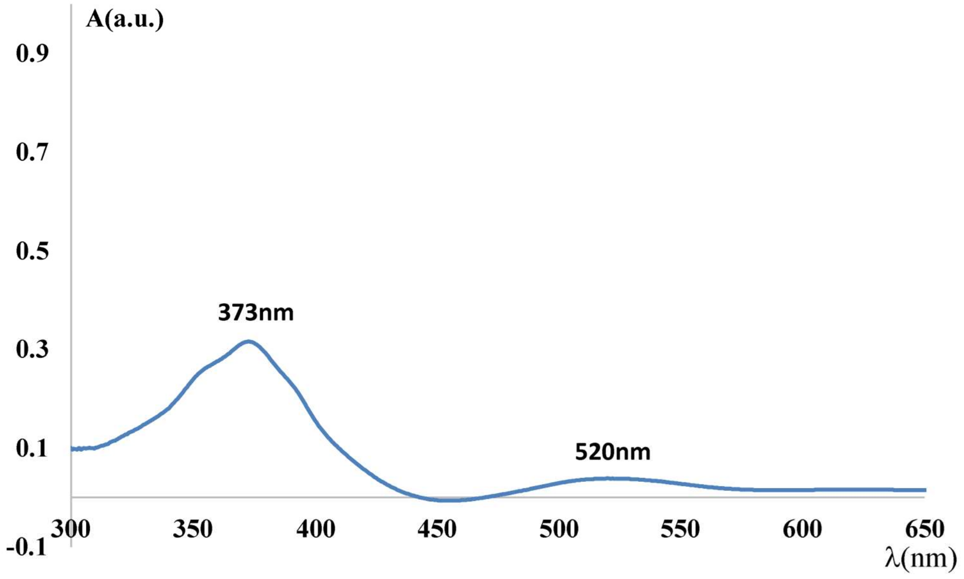

Figure S2. UV-VIS spectra of compound 3 on spun-cast film on ITO from a DCM solution

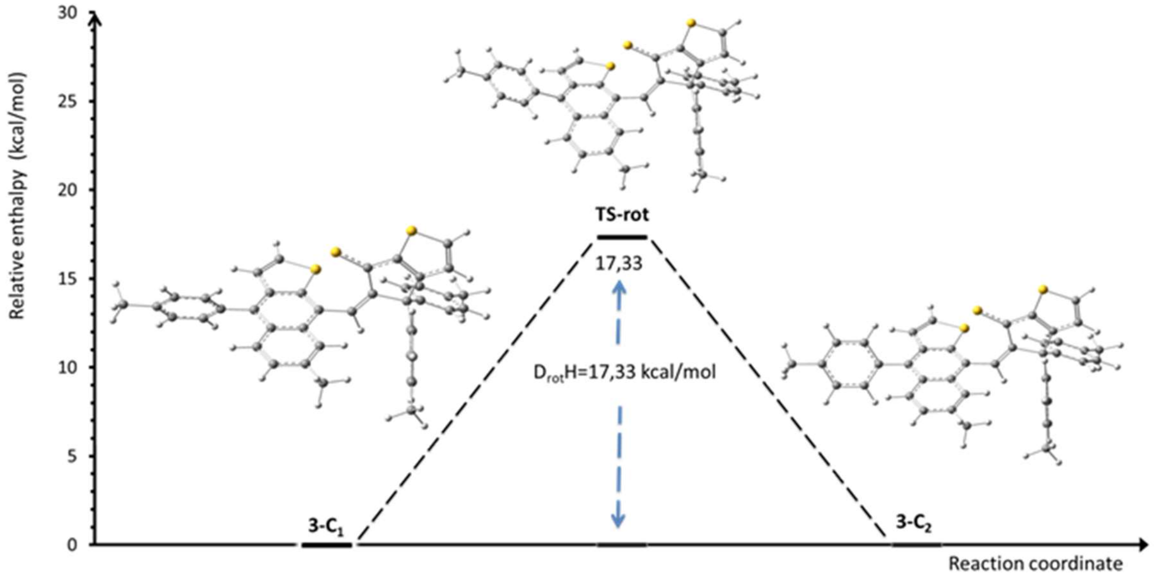

Figure S3. Calculated relative enthalpies profile for the rotation of the phenyl group attached to the naphtho[2,3-*b*]thiophene unit in compound 3

Table S1. Calculated enthalpies for the intermediates involved in the formation of compounds 2 and 3

Species		Enthalpy (Hatree)	Relative enthalpy to rectant (kcal/mol)
**Reactant**	IV-a	-2814,832947	0,00
	IV-b	-2814,835071	0,00
**Transition state**	TS-a	-2814,794167	24,33
	TS-b	-2814,792805	26,52
**Product**	V-a	-2814,801632	19,65
	V-b	-2814,794796	25,27

Table S2. Calculated enthalpies for compounds involved in global transformations 7 to 3 and 7 to 2, respectively

Compound	Enthalpy (Hatree)
**7**	-2967.304186
**3**	-2814.437184
**2**	-2814.411166
**Water**	-76.444452

Total enthalpy balance for the obtaining of **3** (Scheme 3)

Compund **3** + 2xH_2O – Compund **7** = - 0.0219 Hartree (-13.74 kcal/mol)

Total enthalpy balance for the obtaining of **1** (Scheme S1)

Scheme S1: Compund **2** + 2xH_2O – Compund **7** = + 0.0041 Hartree (+2.58 kcal/mol)

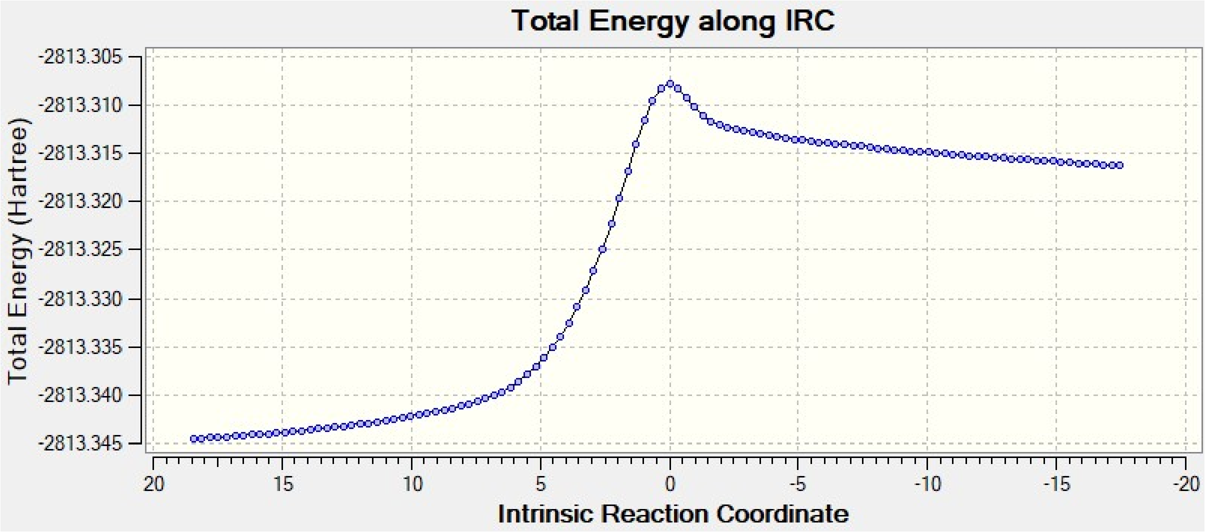

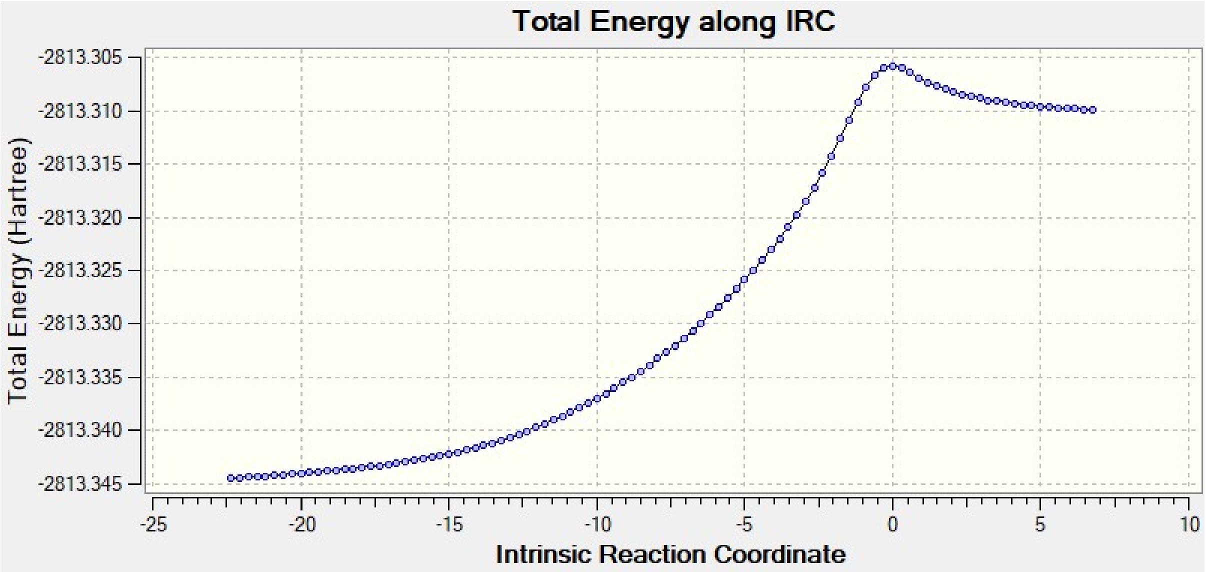

Figure S4. IRC plot for the path a (TS-a connects IV-a and V-a minima) (top) and path b (TS-b connects IV-b and V-b minima, bottom). The IRC calculations were performed at B3LYP-D3/Def2-SVP level of theory, in dichloromethane as solvent, without any symmetry constraints.

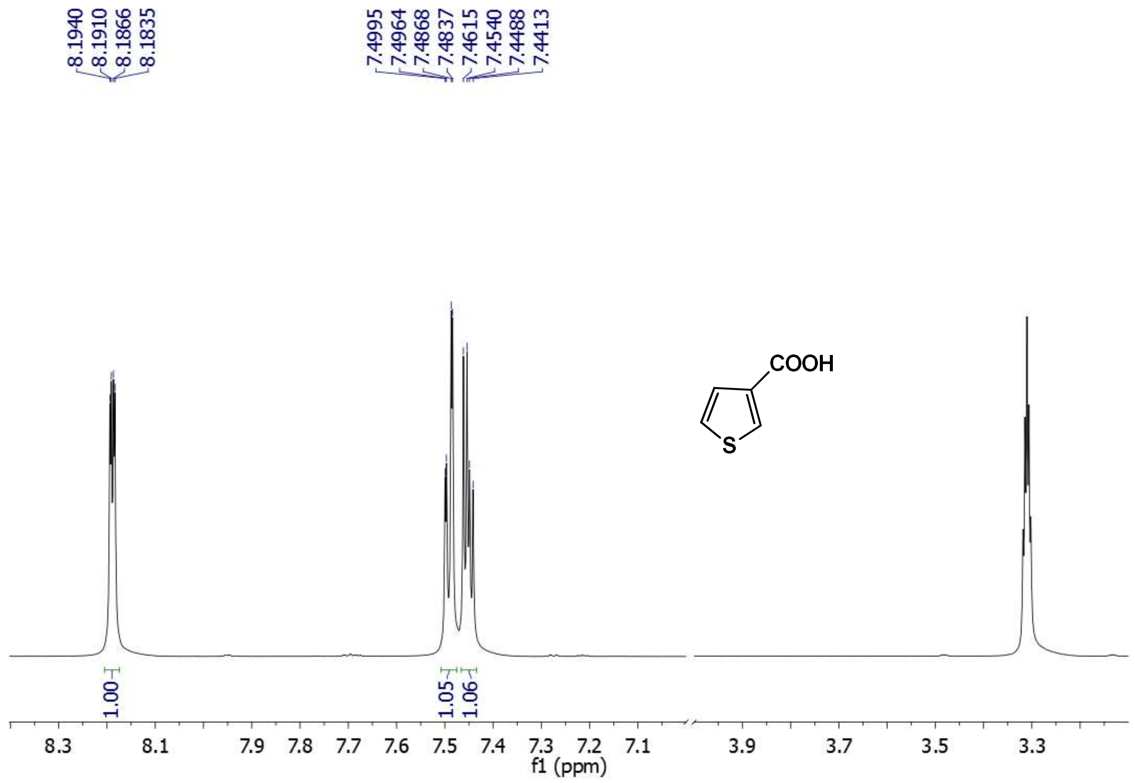

Figure S5. **^{1}H NMR (MeOH-d_4, 400 MHz) spectrum of thiophene-3-carboxylic acid**

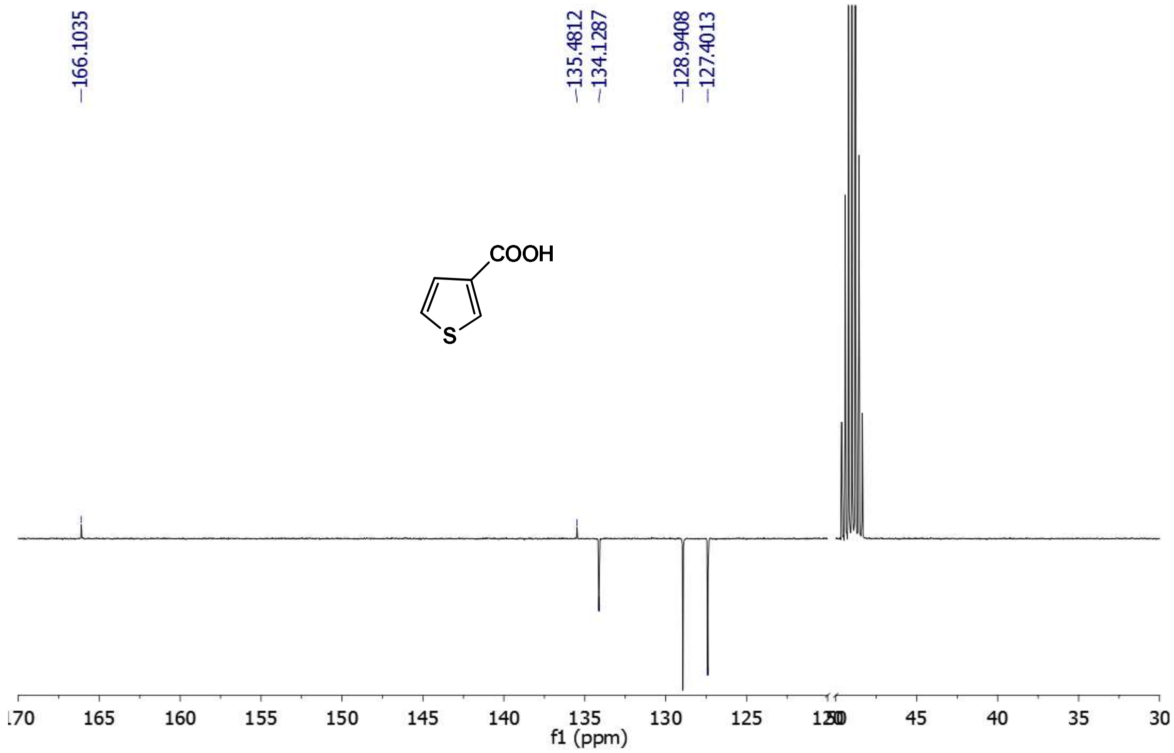

Figure S6. **^{13}C-APT- NMR (MeOH-d_4, 100 MHz) spectrum of thiophene-3-carboxylic acid**

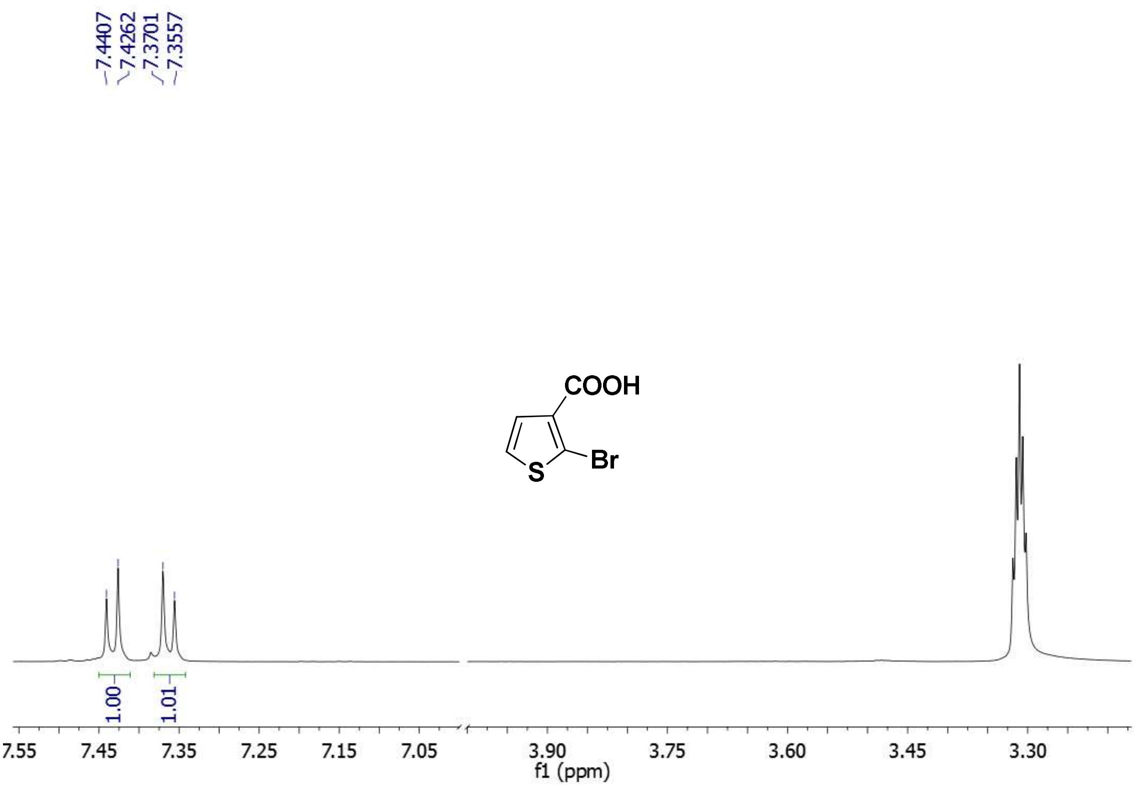

Figure S7. **^{1}H NMR (MeOH-*d*$_4$, 400 MHz) spectrum of 2-bromothiophene-3-carboxylic acid**

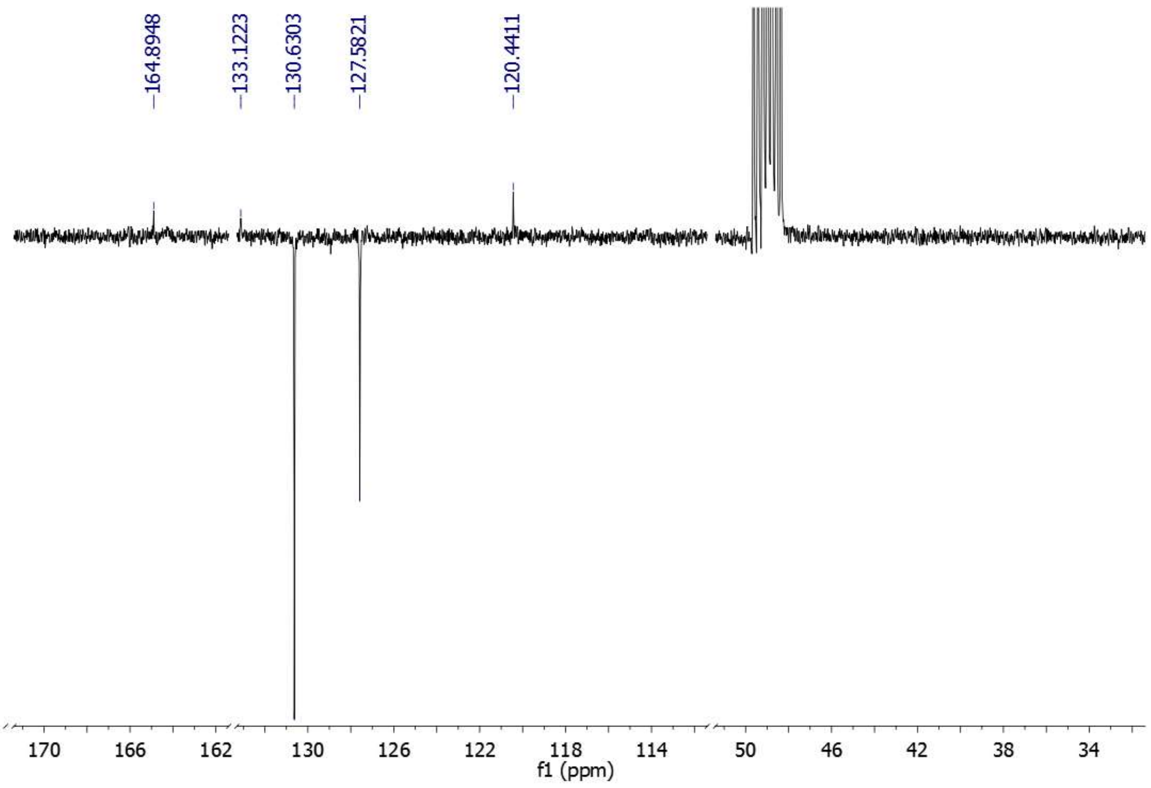

Figure S8. **^{13}C-APT- NMR (MeOH-*d*$_4$, 100 MHz) spectrum of 2-bromothiophene-3-carboxylic acid**

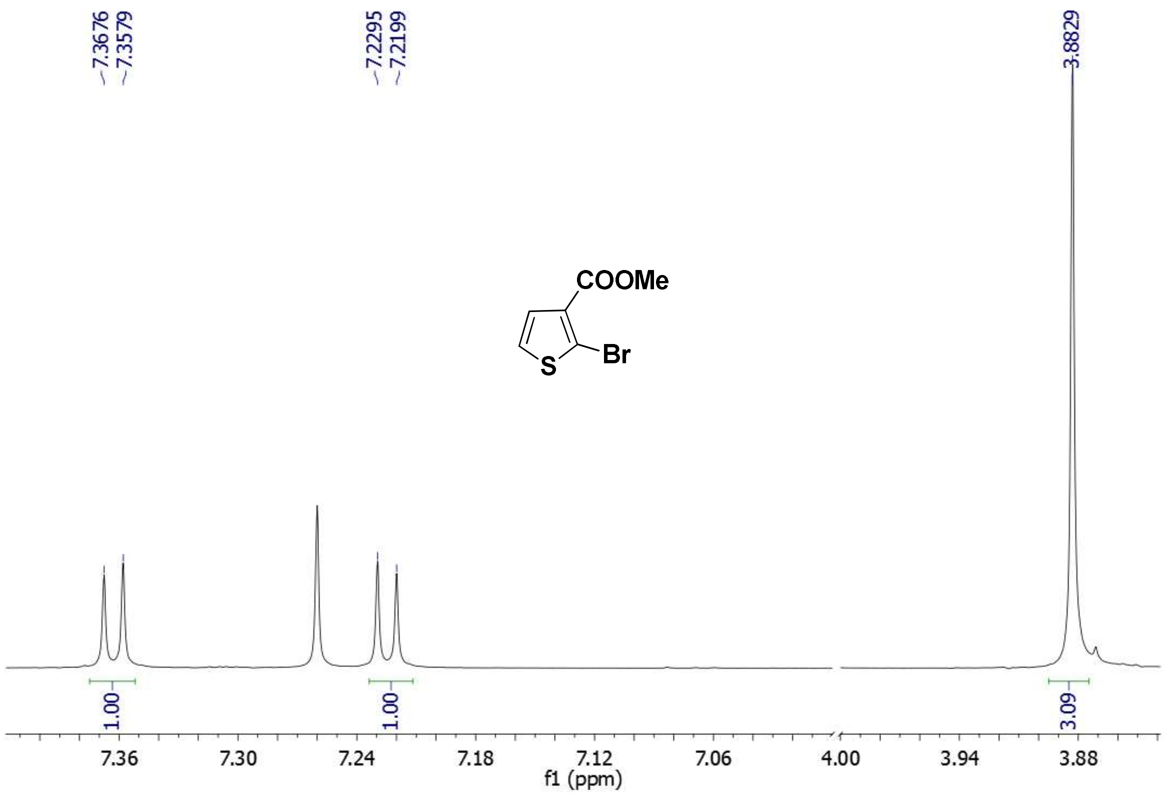

Figure S9. ^{1}H **NMR ($CDCl_3$, 600 MHz) spectrum of compound 5**

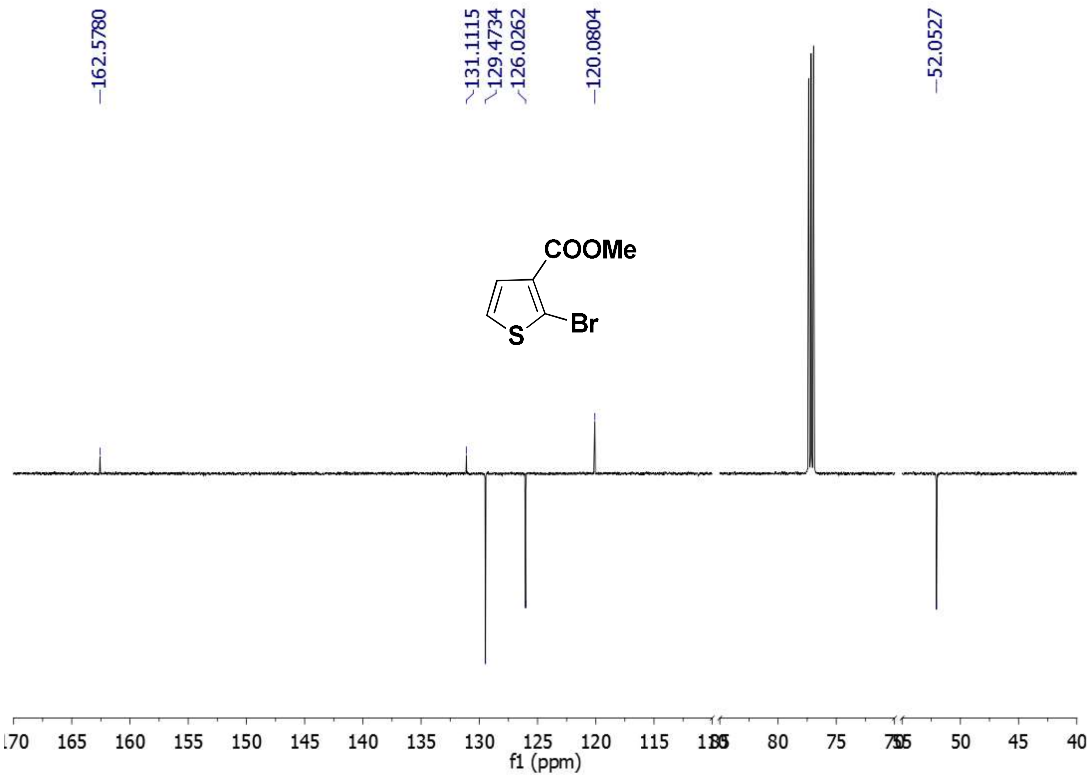

Figure S10. ^{13}C**-APT NMR ($CDCl_3$, 150 MHz) spectrum of compound 5**

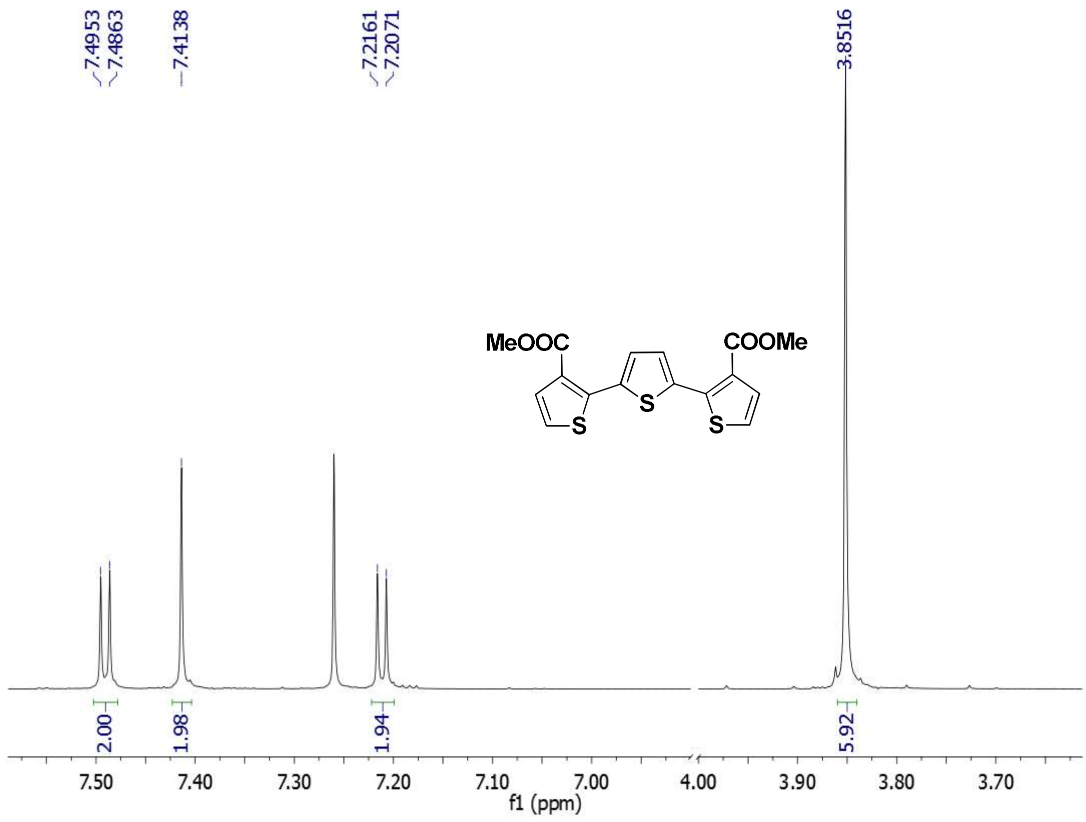

Figure S11. ^{1}H NMR ($CDCl_3$, 600 MHz) spectrum of compound 6

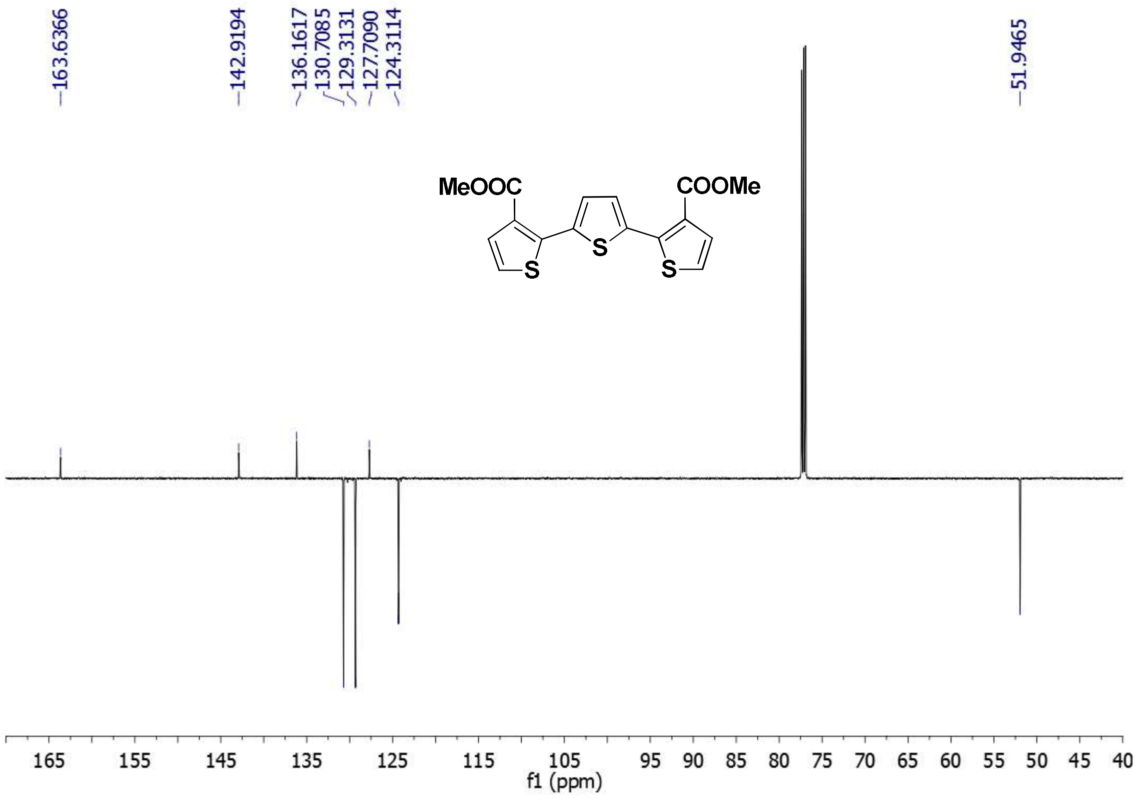

Figure S12. ^{13}C-APT NMR ($CDCl_3$, 150 MHz) spectrum of compound 6

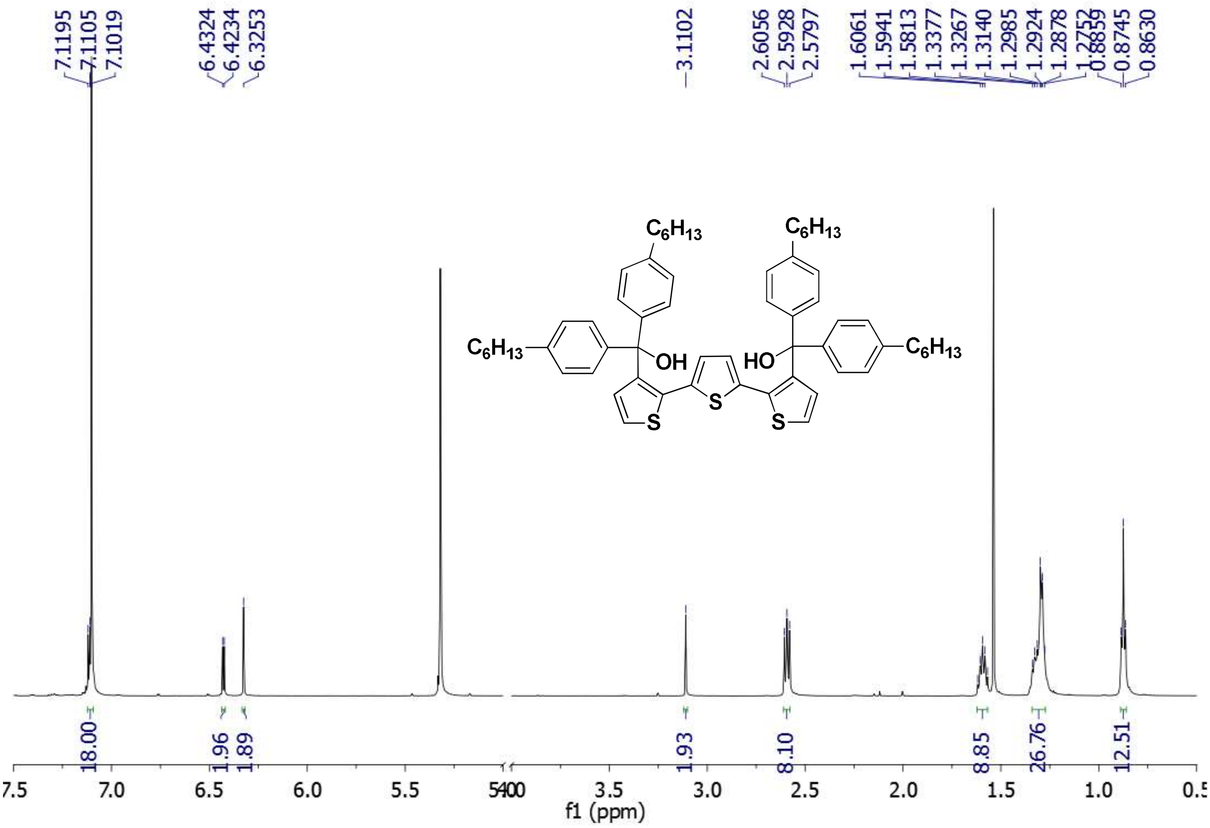

Figure S13. ^{1}H NMR (CD_2Cl_2, 600 MHz) spectrum of compound 7

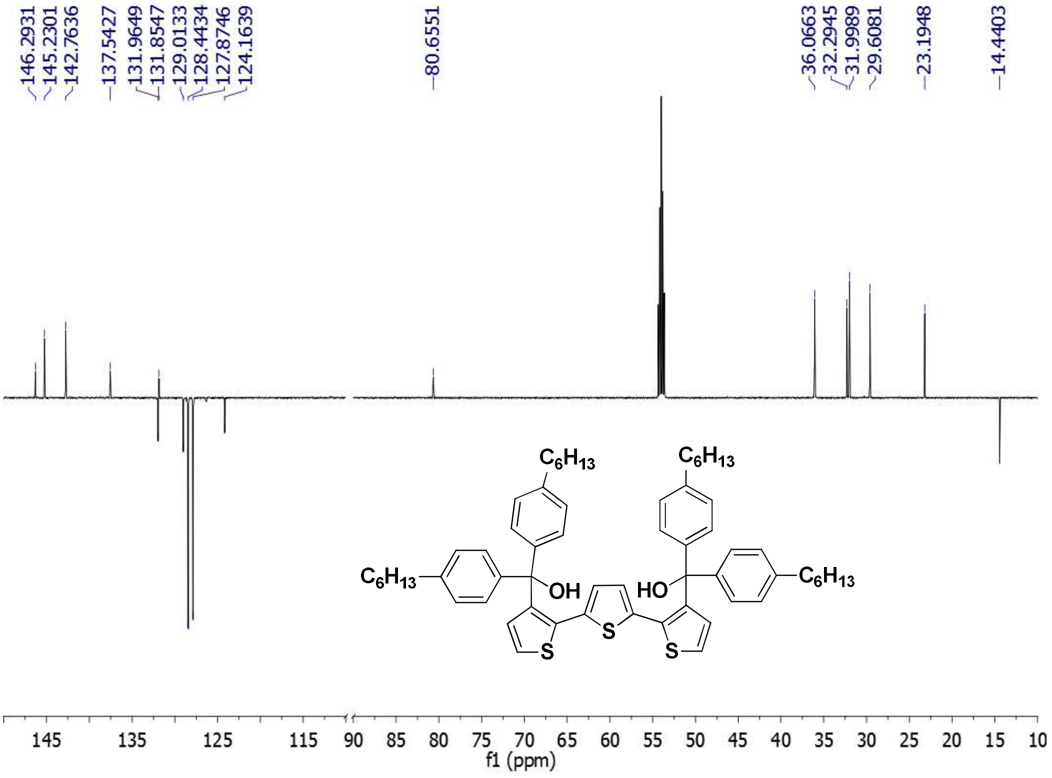

Figure S14. ^{13}C-APT NMR (CD_2Cl_2, 150 MHz) spectrum of compound 7

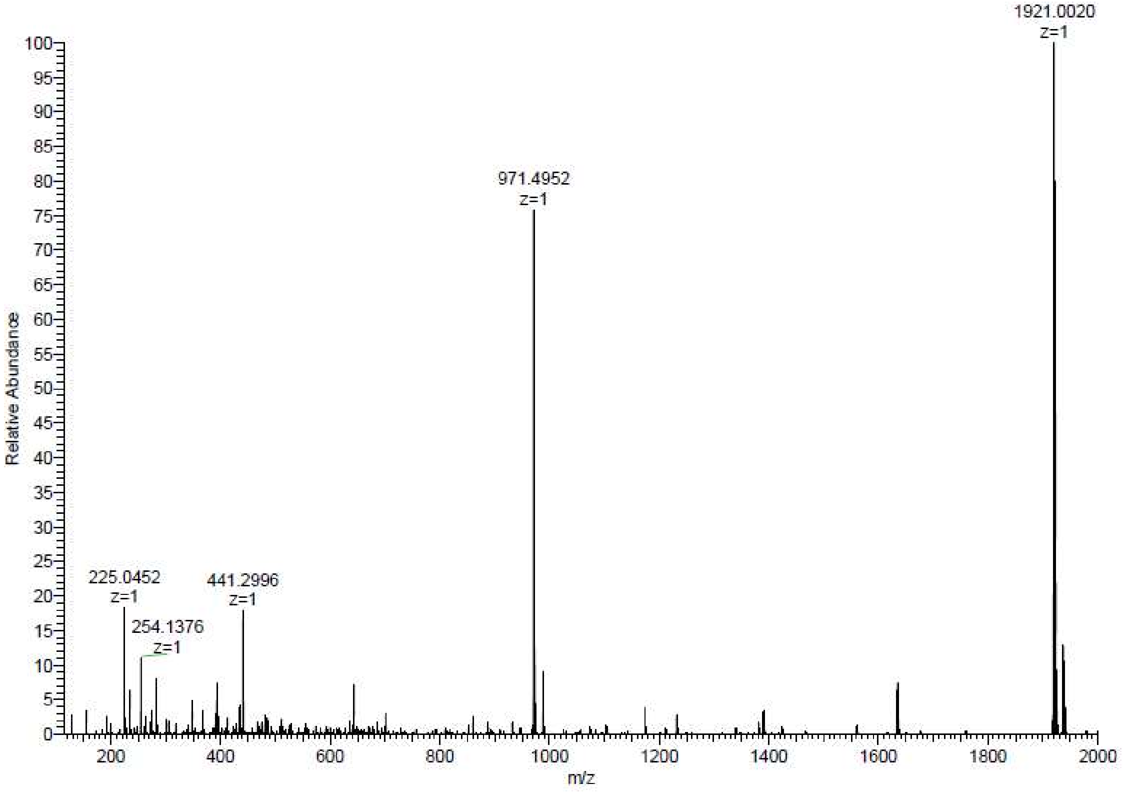

Figure S15. ESI(+)-HRMS spectrum of 7

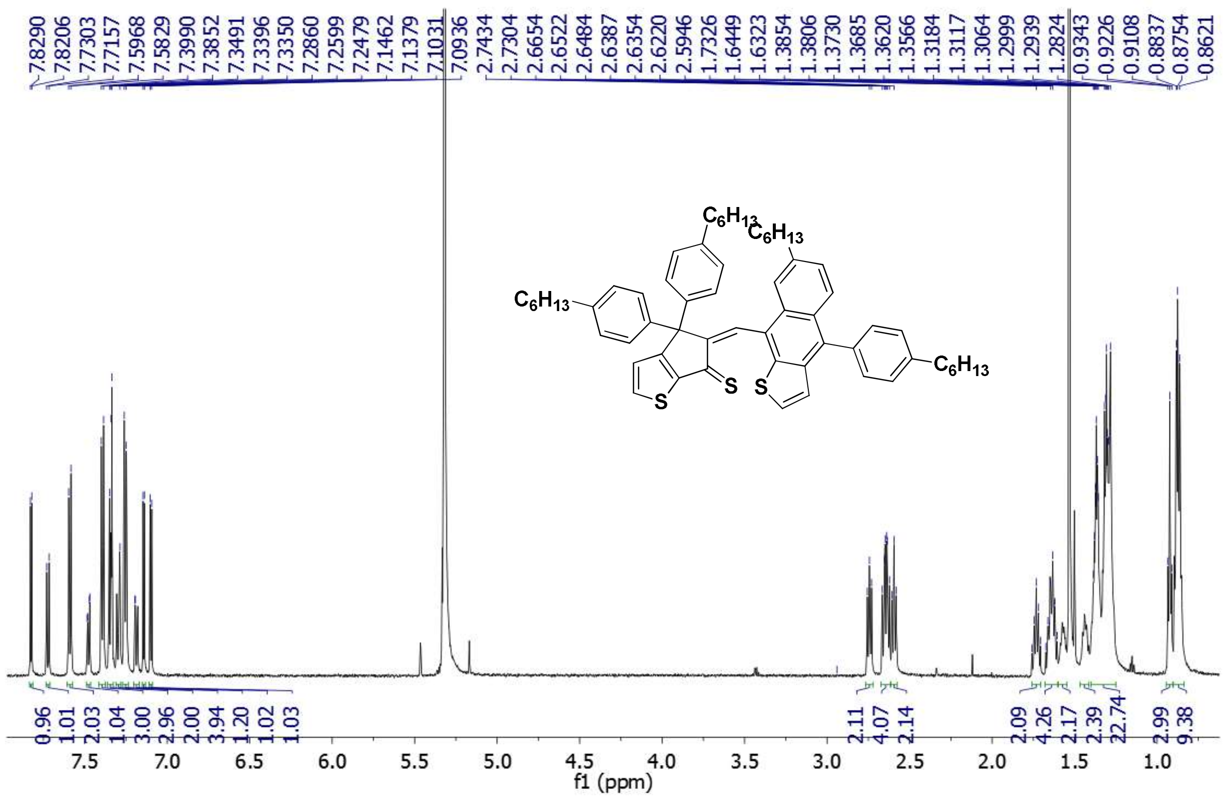

Figure S16. ^{1}H NMR(CD_2Cl_2, 600 MHz) spectrum of compound 3

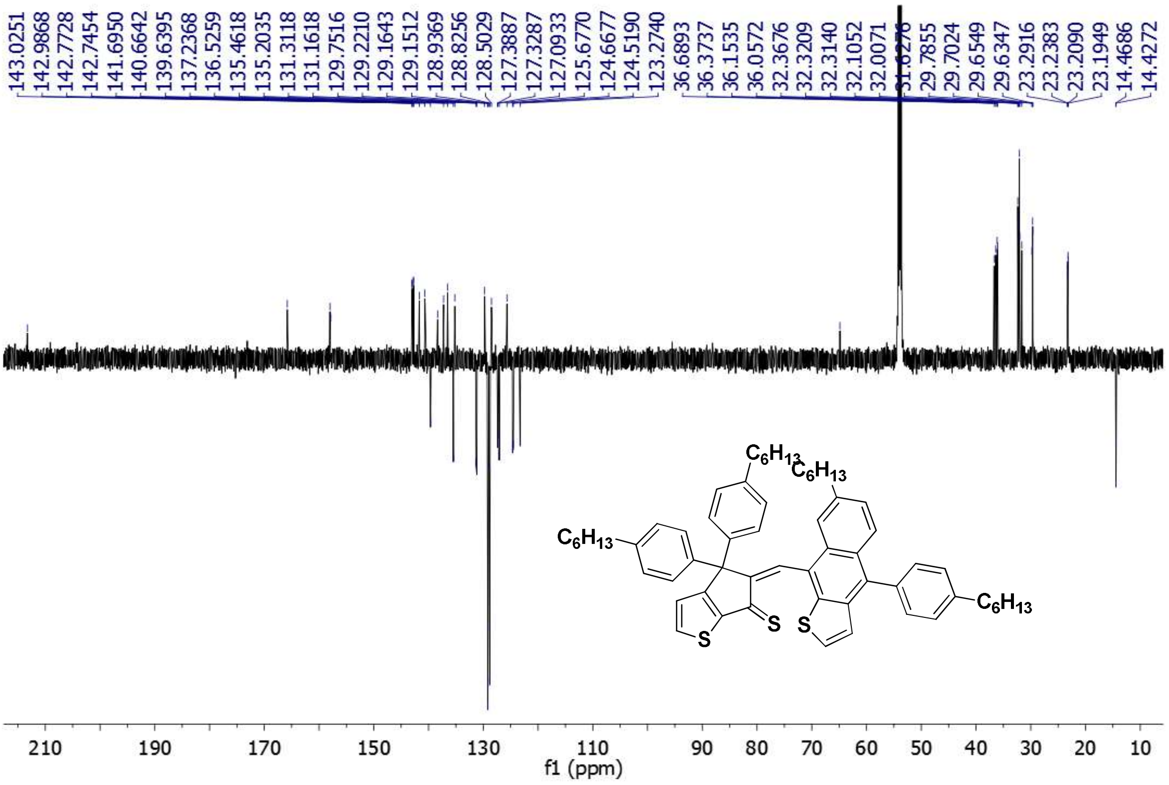

Figure S17. ^{13}**C-APT NMR (**CD_2Cl_2**, 150 MHz) spectrum of compound 3**

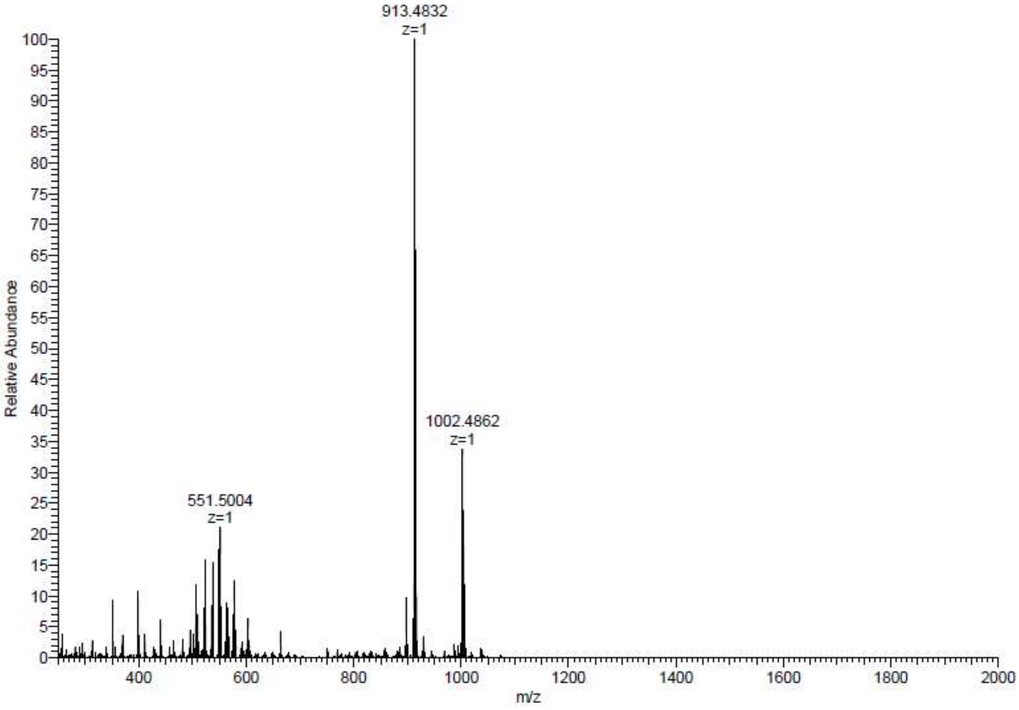

Figure S18. APCI(+)-HRMS spectrum of 3

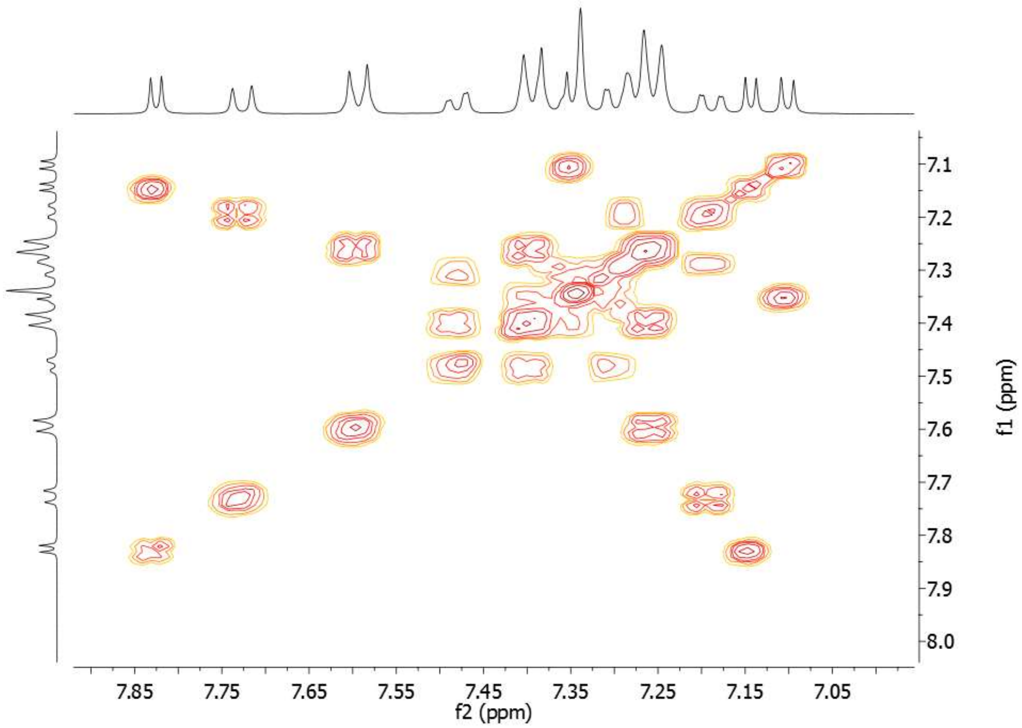

Figure S19. COSY (H,H)-NMR (CD_2Cl_2, 600 MHz) spectrum of compound 3: aromatic region

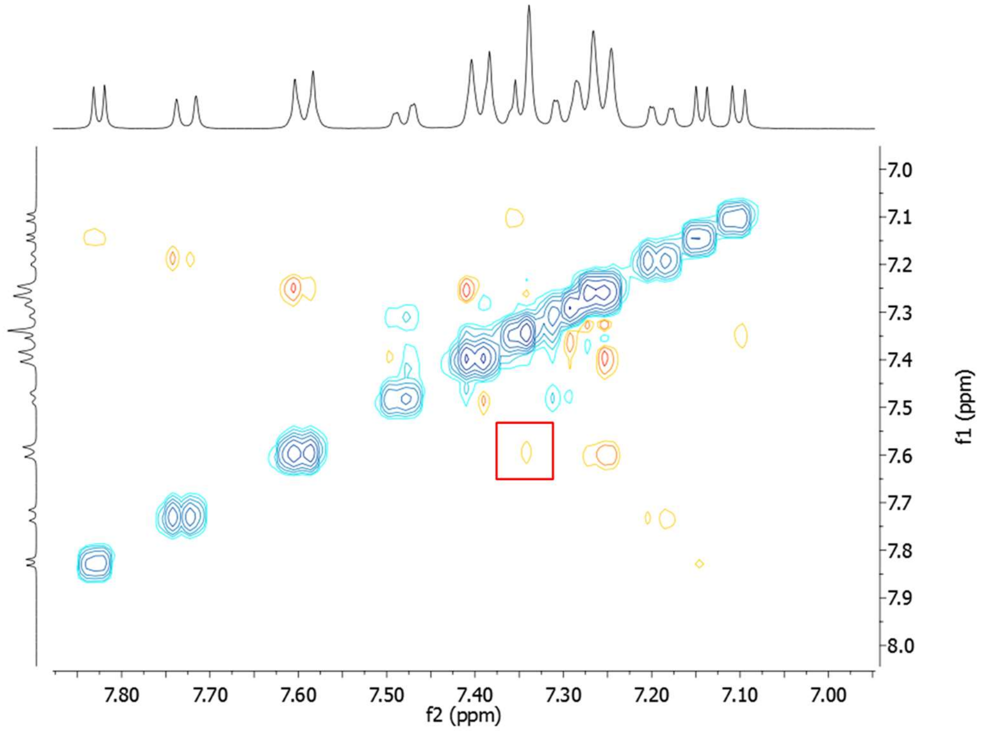

Figure S20. ROESY NMR (CD_2Cl_2, 600 MHz) spectrum of compound 3: aromatic region

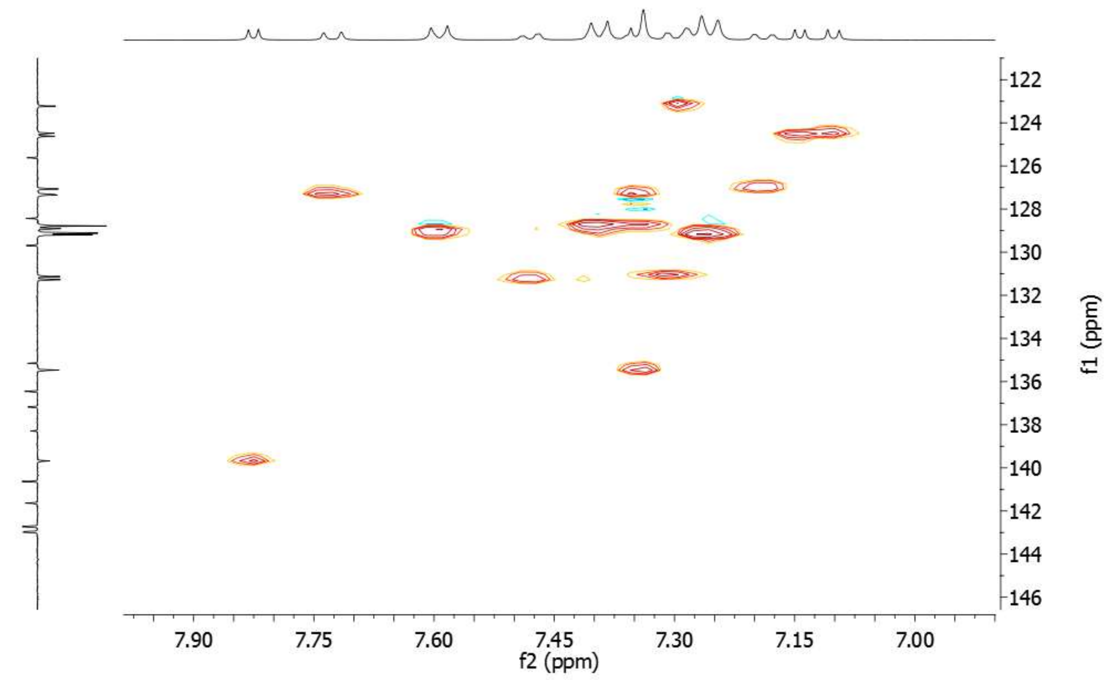

Figure S21. HSQC NMR (CD_2Cl_2, 600 MHz) spectrum of compound 3: aromatic region

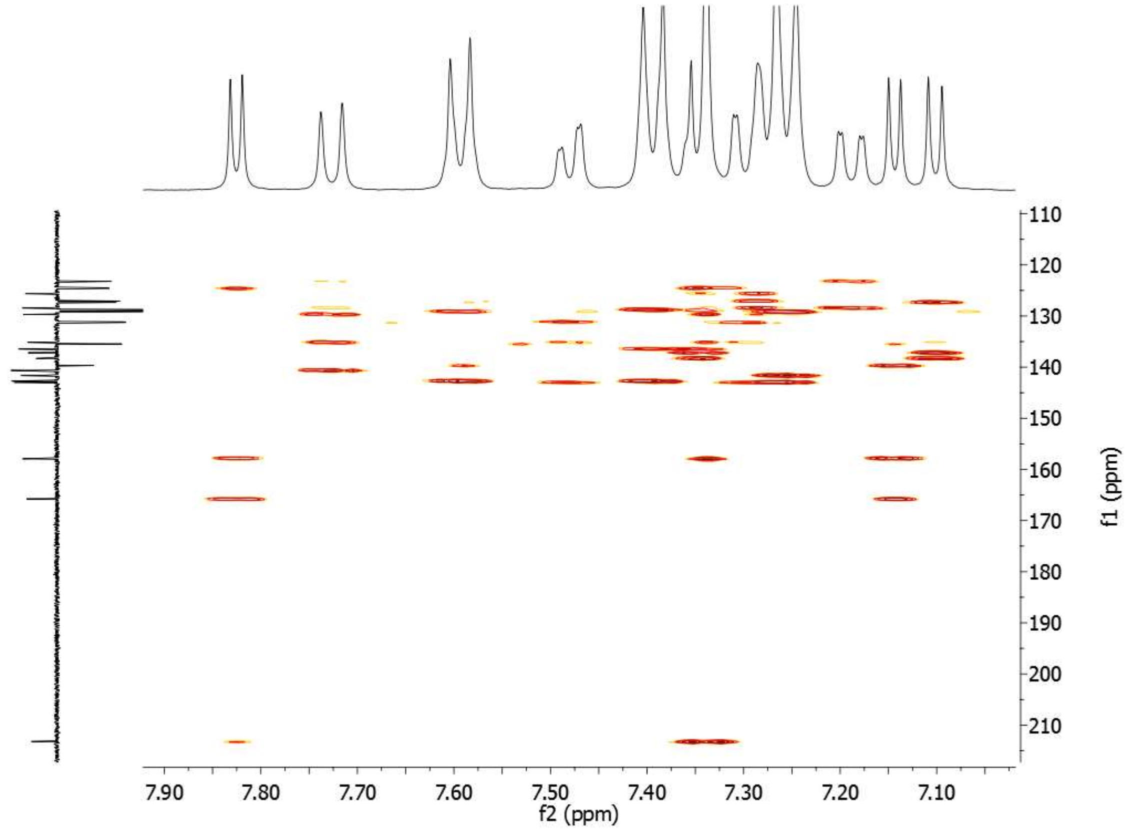

Figure S22. HMBC NMR (CD_2Cl_2, 600 MHz) spectrum of compound 3: aromatic region

Crystal structure determination

The details of the crystal structure determination and refinement for compound **3** are given in Table 1. The crystal was mounted on MiTeGen microMounts cryoloops and data were collected on a Bruker D8 VENTURE diffractometer using Mo-Kα radiation (λ = 0.71073 Å) from a IμS 3.0 microfocus source with multilayer optics, at low temperature (100 K)[1] For structure solving and refinement the Bruker APEX3 Software Package was used. The structure was refined with anisotropic thermal parameters for non-H atoms and hydrogen atoms were placed in fixed, idealized positions and refined with a riding model and a mutual isotropic thermal parameter. The drawings were created using the Diamond program.[1]

Table S3. Crystal data and structure refinement for 3

Empirical formula	$C_{62}H_{72}S_3$	
Formula weight	913.37	
Temperature	100(2) K	
Wavelength	0.71073 Å	
Crystal system	Triclinic	
Space group	P -1	
Unit cell dimensions	a = 10.5801(19) Å	a= 71.903(7)°.
	b = 16.028(4) Å	b= 72.493(6)°.
	c = 17.160(4) Å	g = 77.170(8)°.
Volume	2611.2(9) $Å^3$	
Z	2	
Density (calculated)	1.162 Mg/m^3	
Absorption coefficient	0.180 mm^{-1}	
F(000)	984	
Crystal size	0.090 x 0.080 x 0.040 mm^3	
Theta range for data collection	2.280 to 28.298°.	
Index ranges	-14<=h<=14, -21<=k<=21, -22<=l<=22	
Reflections collected	134242	
Independent reflections	12919 [R(int) = 0.0713]	
Completeness to theta = 25.242°	99.9 %	
Refinement method	Full-matrix least-squares on F^2	
Data / restraints / parameters	12919 / 0 / 593	
Goodness-of-fit on F^2	1.063	
Final R indices [I>2sigma(I)]	R1 = 0.0545, wR2 = 0.1286	
R indices (all data)	R1 = 0.0825, wR2 = 0.1474	
Largest diff. peak and hole	0.983 and -0.530 e.$Å^{-3}$	

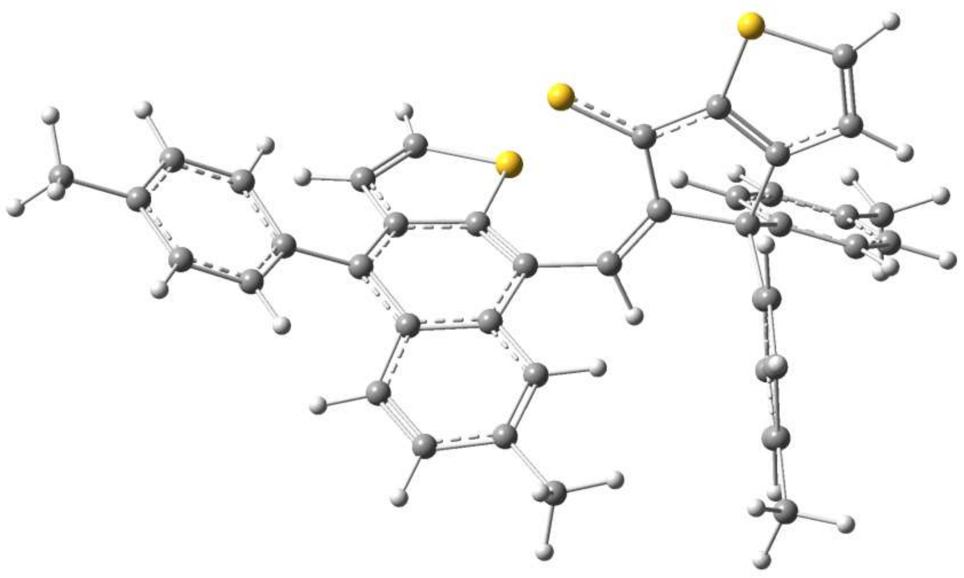

Figure S23. DFT calculated structure of TS-rot

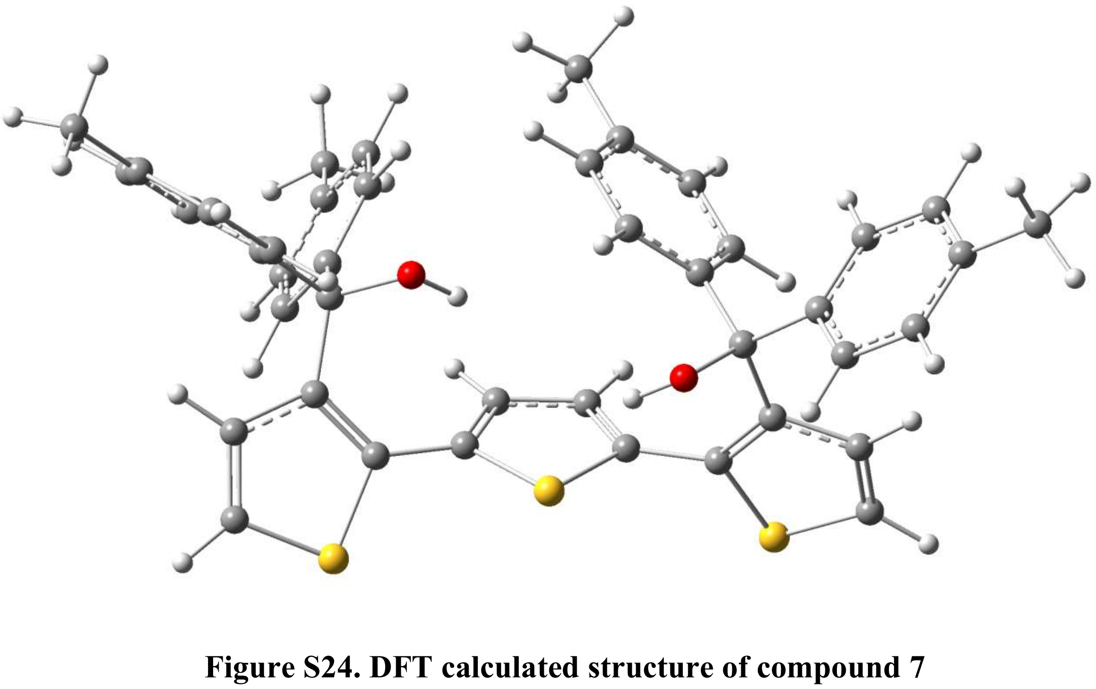

Figure S24. DFT calculated structure of compound 7

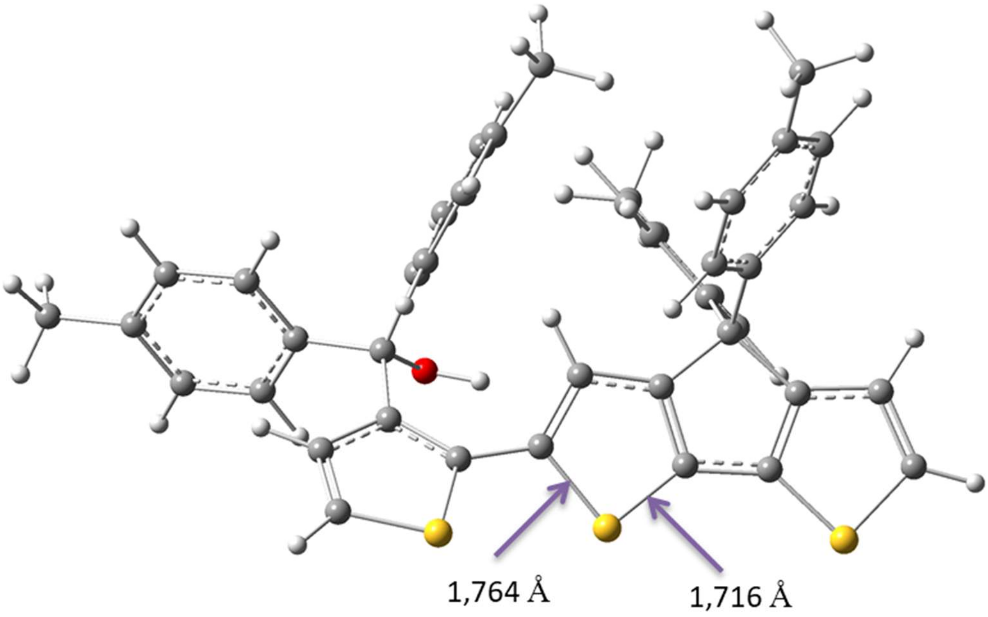

Figure S25. DFT calculated structure of intermediate III

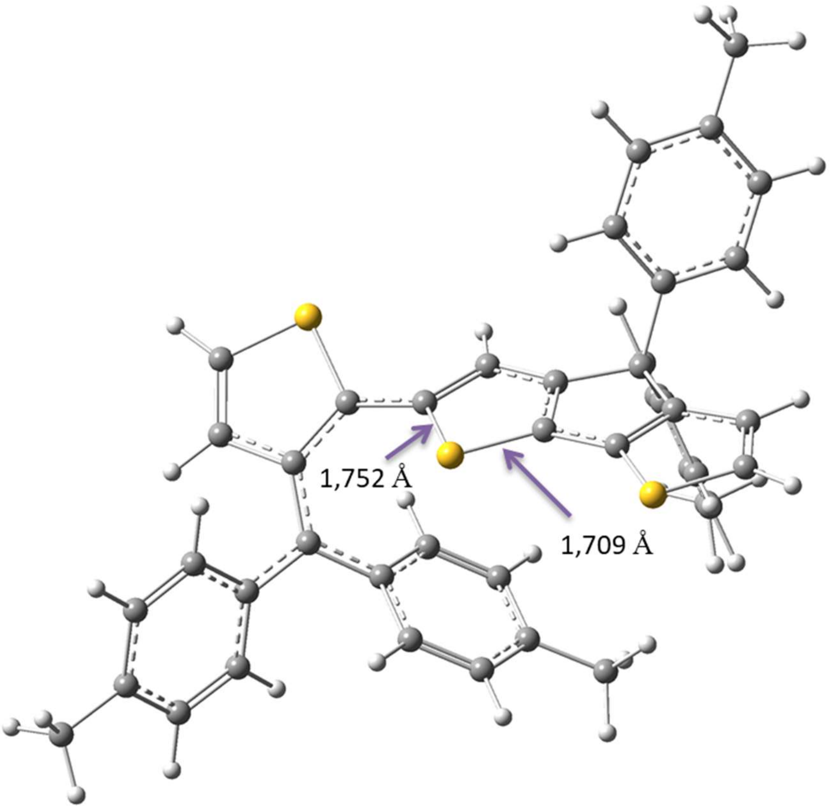

Figure S26. DFT calculated structure of intermediate IV-a

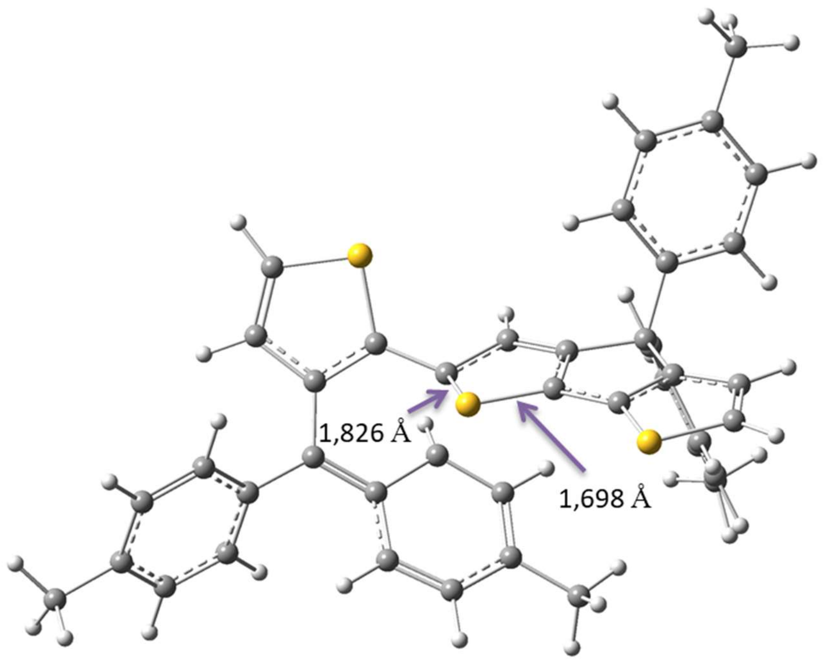

Figure S27. DFT calculated structure of TS-a

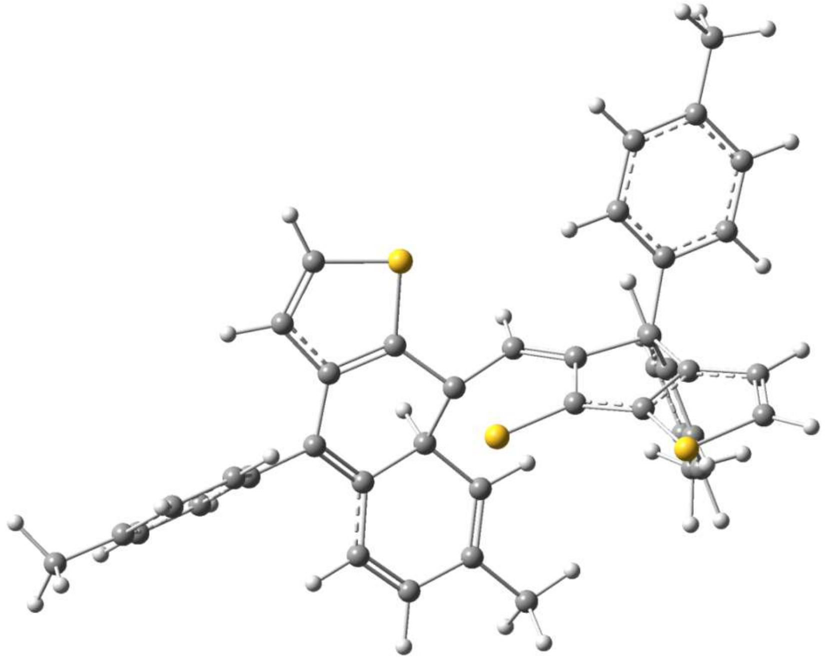

Figure S28. DFT calculated structure of intermediate V-a

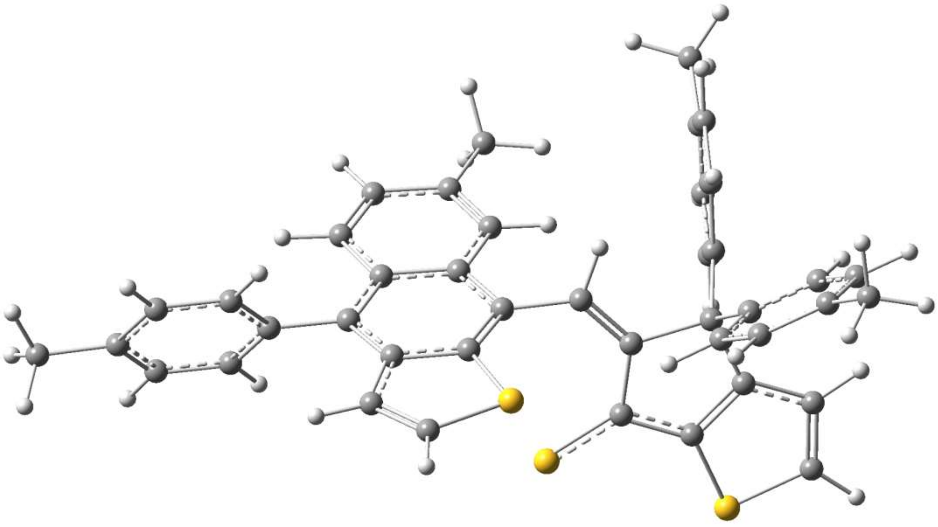

Figure S29. DFT calculated structure of compound 3

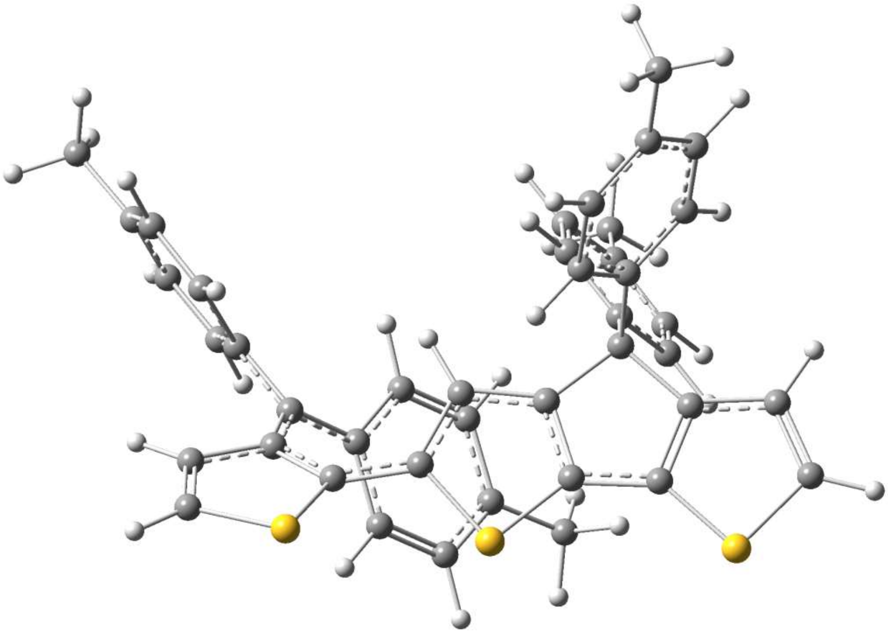

Figure S30. DFT calculated structure of intermediate IV-b

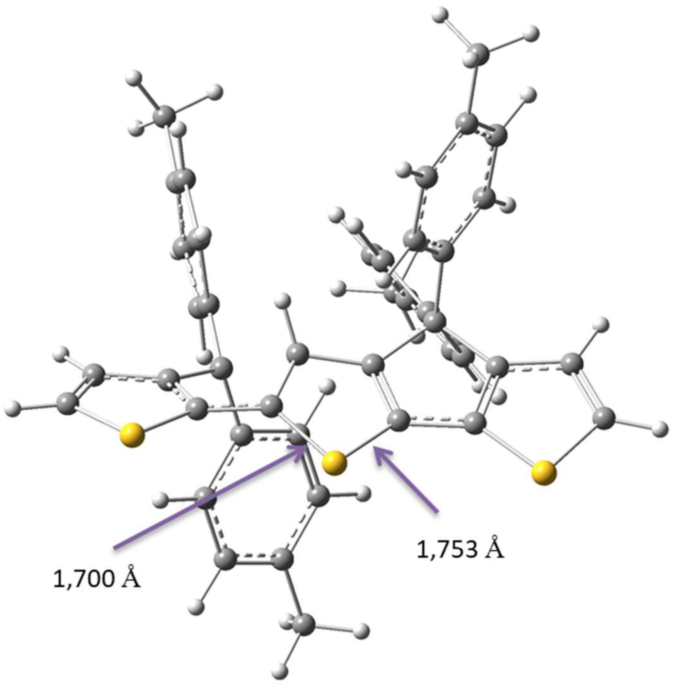

Figure S31. DFT calculated structure of TS-b

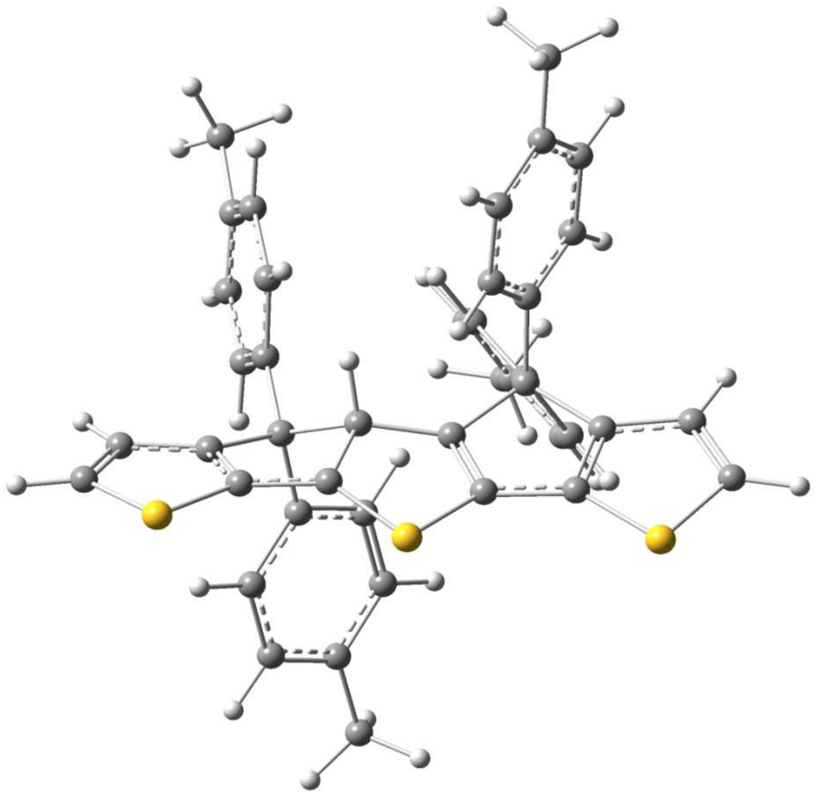

Figure S32. DFT calculated structure of intermediate V-b

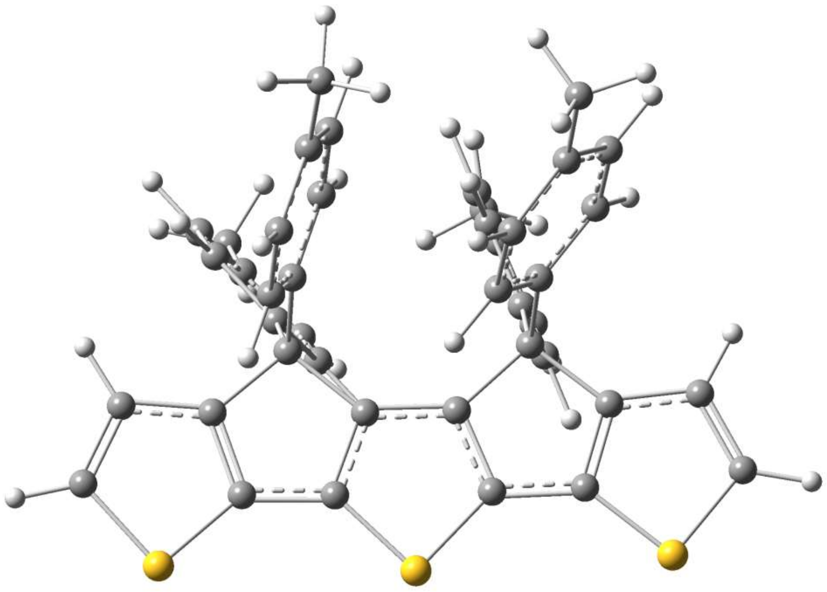

Figure S33. DFT calculated structure of compound 2

Table S4. DFT coordinates of TS-rot

E= -2815.05596861Hatrees

Sum of electronic and thermal Enthalpies= -2814.409565 Hartrees

0 1

S	12.78242000	11.73762300	2.18902000
S	10.31286800	9.10462300	2.05328100
S	11.34911200	5.43697700	2.46949400
C	12.98696000	9.15144500	5.17774600
C	14.24596600	8.27633500	5.24318100
C	15.31168600	8.66565500	6.06050400
H	15.21163900	9.54003800	6.69075500
C	16.49486600	7.94341900	6.08775500
H	17.30147300	8.27049200	6.73392500
C	16.66544100	6.80374500	5.29699200
C	15.60708200	6.42605900	4.47431000
H	15.70698600	5.55171600	3.84165900
C	14.41776400	7.14919100	4.44547700
H	13.62504500	6.82388100	3.78810600
C	17.93986500	6.00488600	5.35604700
H	18.05592400	5.37393500	4.47435100

H	17.94850200	5.35167500	6.23325000
C	13.28918800	10.34790700	4.30224600
C	14.20857700	11.41526900	4.33801400
H	14.95101800	11.57839500	5.10435200
C	14.04504500	12.24079300	3.25357500
H	14.61139700	13.12914500	3.02002700
C	12.45627900	10.38586700	3.20714300
C	11.50153400	9.31749600	3.18132200
C	11.80083100	8.51374800	4.38983500
C	12.45541700	9.55775100	6.56225300
C	12.56546100	8.70644800	7.66158200
H	13.13420900	7.78991900	7.57958900
C	11.94391000	9.01067700	8.86737500
H	12.03857900	8.32200700	9.69892000
C	11.19525200	10.17675100	9.02258700
C	11.09523700	11.03134100	7.92372400
H	10.51676300	11.94402400	8.00817300
C	11.71203600	10.72882900	6.71688400
H	11.59295800	11.40655800	5.88140600
C	10.53574700	10.51419100	10.33289400
H	10.31889100	9.61552600	10.91158400
C	11.11829600	7.48026800	4.89753800
H	11.45926700	7.12114300	5.86503100
C	9.91266800	6.80369400	4.40406800
C	8.71634200	6.88689900	5.16130800
C	8.69651600	7.61270500	6.38435500
H	9.57132700	8.18823700	6.65977600
C	7.62733900	7.58098100	7.24026300
C	6.52806100	6.76379500	6.88210300
H	5.71687600	6.62606700	7.58768500
C	6.47865100	6.12590500	5.67171300
H	5.64810300	5.46644200	5.47970900
C	7.52925800	6.21731700	4.71376100
C	7.48126500	5.60766300	3.40617700
C	8.74045200	5.27991800	2.85239400
C	9.91943400	5.91805900	3.35012800
C	9.10167500	4.34141700	1.81271500
H	8.42315000	3.63548400	1.36579100
C	10.42225400	4.31937800	1.52601100
H	10.92098000	3.66881500	0.82478300
C	6.21319100	5.47296500	2.63197400
C	6.19713200	5.03592100	1.29086500
H	7.11239900	4.79706300	0.78284100
C	5.03362400	4.95326900	0.54212700

H	5.09925000	4.60785400	-0.48338400
C	3.79673100	5.32620400	1.06459700
C	3.80179600	5.84331500	2.35804700
H	2.87843500	6.21233100	2.79000600
C	4.96491600	5.92635400	3.10886000
H	4.88522500	6.41049800	4.06467000
C	2.52874000	5.19292100	0.26899400
H	2.71566800	5.30334600	-0.80021900
H	1.78978600	5.93723300	0.56901800
H	2.07915100	4.20696200	0.42078900
H	9.60142400	11.05598400	10.17839300
H	11.18397200	11.14951800	10.94310800
H	18.81277700	6.65590700	5.42962500
C	7.62496200	8.34013900	8.53753800
H	8.57380800	8.85044600	8.70095300
H	6.82940200	9.09007300	8.54808700
H	7.44418700	7.67155400	9.38312900

Table S5. DFT coordinates of compound 7

E= -2968.00691912 Hartrees

Sum of electronic and thermal Enthalpies= -2967.304186 Hartrees

C	0.05905500	-2.44656700	4.59254100
C	1.01324100	-1.45716300	4.47256600
S	0.16839000	-3.23196000	6.13957100
C	1.80809300	-1.34239100	5.64998900
C	1.47181000	-2.23327300	6.62626900
H	2.61084700	-0.62894000	5.75484900
H	1.92653500	-2.35830100	7.59583300
C	1.20398300	-0.56937700	3.23379500
C	0.14549100	0.53900000	3.18424200
C	-0.52318600	0.98592300	4.32060900
C	-0.14657200	1.15033700	1.96477400
C	-1.46691700	2.00529500	4.23807600
H	-0.31708600	0.53461800	5.28182200
C	-1.08978700	2.16445400	1.88530300
H	0.36617000	0.81915200	1.07288000
C	-1.77276400	2.60953300	3.01985500
H	-1.97488200	2.33065000	5.13853400
H	-1.29890800	2.62081900	0.92515400
C	2.62167900	0.01553600	3.23102400
C	2.89444700	1.31640900	3.63905300

C	3.69351900	-0.79852900	2.85788800
C	4.20241200	1.79449700	3.66943400
H	2.08726100	1.97005000	3.93995600
C	4.99380700	-0.31721000	2.88403300
H	3.50252700	-1.81601900	2.54595100
C	5.27461000	0.99091700	3.28977300
H	4.38570400	2.81269700	3.99271300
H	5.80785700	-0.96937700	2.58823200
C	6.68686000	1.51269700	3.28926000
H	7.00489200	1.77166700	2.27537700
H	7.38627400	0.76346100	3.66474300
H	6.78173500	2.40845400	3.90378400
C	-2.82172700	3.68451400	2.91897800
H	-2.54385200	4.44197000	2.18420900
H	-2.98062100	4.17832400	3.87817100
H	-3.78018300	3.26269600	2.60300200
O	1.13753100	-1.36053700	2.03474600
H	0.23221600	-1.67603200	1.91438100
C	-0.96646500	-2.86857200	3.64398800
C	-1.95340500	-2.12029100	3.05066700
S	-1.06103200	-4.51934200	3.10309800
C	-2.76060700	-2.86083000	2.15162200
H	-2.08885800	-1.06958700	3.25720100
C	-2.40400400	-4.17760200	2.05005400
H	-3.56234200	-2.43209000	1.56940200
C	-2.98296600	-5.20995300	1.18974700
C	-2.53453800	-5.68636500	-0.02619500
S	-4.46455800	-5.97812800	1.65466300
C	-3.41568800	-6.66722500	-0.56569000
C	-1.25833400	-5.18183000	-0.72375700
C	-4.49604800	-6.92465800	0.22805200
H	-3.25370800	-7.15455800	-1.51435900
C	-1.04807000	-5.91259400	-2.05768900
C	-1.32783700	-3.66071900	-0.91804000
O	-0.10767000	-5.53410500	0.06463000
H	-5.30191900	-7.61742700	0.04544400
C	-0.48029400	-7.18938800	-2.05353100
C	-1.44823900	-5.38098600	-3.27964100
C	-2.52720100	-3.02816700	-1.25190600
C	-0.18928900	-2.87425100	-0.77955600
H	-0.15704800	-5.07184900	0.91235800
C	-0.31668600	-7.90180800	-3.23274900
H	-0.16867600	-7.62772600	-1.11619800
C	-1.28602000	-6.10062300	-4.46046900

H	-1.89091500	-4.39637700	-3.32635200
C	-2.58276800	-1.65542600	-1.43511000
H	-3.43226500	-3.61292400	-1.35406800
C	-0.25035000	-1.49381600	-0.95333300
H	0.75359800	-3.33680000	-0.52340600
C	-0.71567400	-7.37131200	-4.46206200
H	0.12631900	-8.89074900	-3.19751200
H	-1.60829200	-5.65829100	-5.39614000
C	-1.44430500	-0.85885300	-1.28506900
H	-3.52903400	-1.19105000	-1.68897700
H	0.64997900	-0.90758900	-0.81817300
C	-0.50755500	-8.13767000	-5.74104700
C	-1.52181100	0.63138100	-1.48037100
H	-1.17000800	-7.78259400	-6.53120700
H	-0.68530000	-9.20490200	-5.59760000
H	0.52039900	-8.02578200	-6.09760100
H	-0.55780800	1.10874300	-1.30539500
H	-2.24845300	1.07785900	-0.79836100
H	-1.83941200	0.87682200	-2.49671100

Table S6. DFT coordinates of intermediate III

E= -2891.53175719 Hartrees

Sum of electronic and thermal Enthalpies= -2890.857136 Hartrees

C	2.01159700	-0.70485200	-2.30656300
C	1.05318800	-0.22919400	-1.44022800
C	0.42994800	-1.23512900	-0.68594400
C	0.90012500	-2.49926300	-0.98448100
S	2.13367900	-2.41559000	-2.24282500
C	2.60620300	0.41111600	-2.99845900
C	2.00208800	1.57911300	-2.57598100
S	3.84055500	0.70622600	-4.15261500
C	2.54465800	2.73001500	-3.18876400
C	3.54720900	2.41292600	-4.06828500
H	2.22245100	3.74384000	-3.00155000
H	4.13574700	3.08286400	-4.67423200
C	0.90055000	1.28566700	-1.55667500
C	-0.51484400	1.55402000	-2.10004100
C	-0.76923600	1.80651300	-3.44388200
C	-1.60501800	1.45829300	-1.23082500
C	-2.07558400	1.96073500	-3.90459200
H	0.05031500	1.87986800	-4.14572900

C	-2.90200300	1.60621000	-1.69347800
H	-1.43798300	1.25775700	-0.18127300
C	-3.16469500	1.86394500	-3.04254200
H	-2.24357600	2.15779900	-4.95713500
H	-3.72750600	1.51582800	-0.99648500
C	1.13979000	2.04189400	-0.24598000
C	0.84736100	3.40656100	-0.17619100
C	1.64233100	1.42367000	0.89515600
C	1.03926100	4.12023600	0.99708400
H	0.44616400	3.91241600	-1.04550800
C	1.82875400	2.14161500	2.07363700
H	1.88427700	0.37021000	0.87932800
C	1.52495300	3.49837000	2.15102100
H	0.79997500	5.17729200	1.02128800
H	2.21033800	1.62845700	2.94881000
H	-0.29301900	-1.04551300	0.08742600
C	0.55090400	-3.77385600	-0.37872300
C	-0.65608100	-4.24778400	0.11587200
S	1.81140100	-4.95504300	-0.14470600
C	-0.52428500	-5.55057100	0.67518800
C	-1.99176500	-3.48706500	0.08959600
C	0.73745700	-6.06167700	0.59813400
H	-1.35049400	-6.09260900	1.10810700
C	-1.99516000	-2.34573500	1.11833100
C	-3.15668200	-4.45259400	0.33109000
O	-2.22628900	-2.95145300	-1.22638500
H	1.08553700	-7.02660700	0.92966100
C	-1.12950700	-2.31851700	2.20960400
C	-2.85661500	-1.26503200	0.93440000
C	-3.74735100	-4.60152300	1.58169000
C	-3.61205200	-5.25835600	-0.71407400
H	-1.49828400	-2.35791100	-1.45478300
C	-1.07889500	-1.21268800	3.05108900
H	-0.45627700	-3.14693100	2.38479100
C	-2.80440200	-0.16308100	1.77726200
H	-3.53942700	-1.27003900	0.09610700
C	-4.76713000	-5.52771100	1.78316300
H	-3.41292500	-3.99629800	2.41324100
C	-4.63266800	-6.17592100	-0.51108300
H	-3.15928000	-5.16368800	-1.69106600
C	-1.89498900	-0.10232400	2.83518000
H	-0.37376300	-1.20487800	3.87413700
H	-3.46542200	0.67644400	1.59503500
C	-5.23202600	-6.32848000	0.74220000

H	-5.20760500	-5.62343600	2.76889800
H	-4.96823200	-6.78896700	-1.33996900
C	-1.75845700	1.13824700	3.67561100
H	-1.47092100	0.89796900	4.70018700
H	-0.98229500	1.79004800	3.26272800
H	-2.68786400	1.70837500	3.70213800
C	1.67615600	4.26355600	3.43851800
H	2.00544900	5.28784000	3.25634800
H	0.72160700	4.31920200	3.97004800
H	2.39501100	3.78520600	4.10461600
C	-4.57756400	2.04402400	-3.53028600
H	-4.99353600	2.99334600	-3.18174800
H	-4.62767600	2.03886600	-4.61935200
H	-5.22788100	1.25105900	-3.15505200
C	-6.35861300	-7.30604500	0.94682300
H	-6.47362900	-7.56793100	1.99917000
H	-7.30755500	-6.88132900	0.60701200
H	-6.19471200	-8.22441600	0.38034800

Table S7. DFT coordinates of intermediate IV-a

E= -2815.49147049 Hartrees

Sum of electronic and thermal Enthalpies= -2814.832947 Hartrees

C	1.10126800	1.14213600	-0.60478900
C	0.28334800	1.53272400	0.44502000
C	0.18201200	0.56535400	1.44707900
C	0.89706300	-0.58278600	1.14516000
S	1.75031700	-0.42275500	-0.37711700
C	1.11159100	2.17389100	-1.60070400
C	0.26955900	3.19454800	-1.19947400
S	1.85030400	2.50252400	-3.11411400
C	0.22485000	4.26126700	-2.12089100
C	1.02720500	4.02322000	-3.20750800
H	-0.36295000	5.15958200	-2.00416900
H	1.18600000	4.65837500	-4.06432600
C	0.92017800	-1.79732000	1.91101400
C	0.88689500	-3.14800200	1.48672100
S	1.11902000	-1.70559200	3.61709600
C	1.06159000	-4.05150000	2.59859400
C	1.14575500	-3.42537800	3.79298300
H	1.03493500	-5.12451200	2.49037700
H	1.20791000	-3.86851200	4.77372600

C	-0.37539200	2.88077800	0.15108800
C	-1.88947200	2.62983900	0.06059900
C	-2.54300200	2.44944000	-1.15378000
C	-2.63227800	2.48312100	1.23533300
C	-3.89846500	2.13340200	-1.19351700
H	-1.99713000	2.54890700	-2.08206200
C	-3.97961000	2.16014000	1.19176000
H	-2.15159700	2.62691000	2.19474200
C	-4.64154700	1.97917000	-0.02656500
H	-4.38084400	2.00100000	-2.15439500
H	-4.52928000	2.04780700	2.11927600
C	-0.02463900	3.98896100	1.14865100
C	-0.74599500	5.18488300	1.13494400
C	1.04656000	3.87869500	2.03064700
C	-0.40634200	6.23148400	1.97930700
H	-1.58657600	5.29661200	0.46173200
C	1.38326600	4.93068800	2.87772600
H	1.63065000	2.96849500	2.06083000
C	0.66416400	6.12421100	2.87119000
H	-0.98299000	7.14871800	1.94602000
H	2.22189800	4.81627000	3.55478700
C	1.00489200	7.25102700	3.80947100
H	0.86983200	8.22149200	3.32906500
H	0.35650100	7.23256500	4.69010800
H	2.03559100	7.18142400	4.15851600
C	-6.11112600	1.65654600	-0.06767200
H	-6.39945700	1.23416000	-1.03071100
H	-6.38246800	0.94538600	0.71454200
H	-6.71162500	2.55648900	0.09149500
H	-0.43463200	0.64589500	2.32997200
C	0.57572300	-3.62175900	0.17343300
C	1.15507400	-4.87160300	-0.26926700
C	2.30441200	-7.30117100	-1.13360500
C	0.43252800	-5.76607800	-1.08613800
C	2.46838900	-5.22639800	0.10297100
C	3.03092000	-6.40702900	-0.33644300
C	0.99543000	-6.95832800	-1.49405000
H	-0.58696200	-5.53306900	-1.35942800
H	3.05398100	-4.54230700	0.70155500
H	4.05235000	-6.64345800	-0.06645600
H	0.41452800	-7.64164800	-2.10052000
C	-0.34743600	-2.91752100	-0.68993000
C	-2.07255600	-1.45667100	-2.38114500
C	-0.17693500	-2.93253300	-2.08956700

C	-1.01774600	-2.20920700	-2.91013200
C	-2.26212300	-1.46038100	-0.99206400
H	0.65765400	-3.46821500	-2.51897600
H	-0.85113400	-2.21355300	-3.97999800
H	-3.08095400	-0.89467800	-0.56597100
C	-2.95918200	-0.65092900	-3.28094500
H	-3.93024300	-0.46504200	-2.82490200
H	-2.50032800	0.32166200	-3.48054900
H	-3.10423300	-1.14617600	-4.24146100
C	2.90200000	-8.60632800	-1.56528600
H	2.49844800	-8.93214300	-2.52408200
H	3.98746800	-8.54361200	-1.63993200
H	2.66823800	-9.38357800	-0.83130300
C	-1.42074900	-2.17137400	-0.16268800
H	-1.59654900	-2.17500300	0.90358000

Table S8. DFT coordinates of TS-a

E= -2815.45118838 Hartrees

Sum of electronic and thermal Enthalpies= -2814.794167 Hartrees

C	-1.18257900	-0.53685200	-3.04195200
C	-2.05566000	-0.26710900	-1.95909800
C	-2.20624400	-1.32591800	-1.11627500
C	-1.54287300	-2.52395200	-1.56249400
S	-0.45156700	-2.06692300	-2.95336400
C	-1.18325500	0.57630700	-3.91706100
C	-2.03070400	1.55818800	-3.41287900
S	-0.41854100	1.06491200	-5.37766800
C	-2.05161600	2.71564600	-4.20768900
C	-1.23114400	2.58410000	-5.30339200
H	-2.63131100	3.60303300	-4.00409900
H	-1.05921100	3.31129100	-6.08151900
C	-1.03411800	-3.52458700	-0.59731000
C	-1.07676600	-4.90720300	-0.74806600
S	-0.25761800	-3.07666400	0.86247700
C	-0.49705600	-5.57645200	0.37422600
C	-0.01476100	-4.71938300	1.31377500
H	-0.45731100	-6.65065600	0.46952200
H	0.44956900	-4.95805800	2.25691800
C	-2.69115000	1.11687200	-2.10898800
C	-4.21154000	0.93435100	-2.22357700
C	-4.87748100	0.96443200	-3.44303000

C	-4.95160600	0.64492000	-1.07360600
C	-6.24581200	0.71208200	-3.51353300
H	-4.33562100	1.17364100	-4.35503200
C	-6.31087400	0.38825900	-1.14883100
H	-4.45986000	0.62667800	-0.10922700
C	-6.98713700	0.41740300	-2.37319000
H	-6.73821800	0.74180200	-4.47828900
H	-6.85980500	0.16303300	-0.24186300
C	-2.30300600	2.10076200	-0.99671900
C	-3.00113100	3.30310500	-0.86923400
C	-1.22163900	1.87319000	-0.15023500
C	-2.63044100	4.24254600	0.08147300
H	-3.84772200	3.50422900	-1.51323100
C	-0.85501800	2.81764300	0.80352800
H	-0.65352600	0.95587400	-0.22700800
C	-1.55166500	4.01684800	0.94041300
H	-3.18991100	5.16755000	0.15903400
H	-0.01103100	2.61310500	1.45177500
C	-1.17811000	5.02407400	1.99444800
H	-1.31206800	6.04466700	1.63263800
H	-1.80866300	4.90876700	2.88058900
H	-0.14147000	4.90449700	2.31063200
C	-8.46797900	0.15908300	-2.44008400
H	-8.79895900	-0.00213500	-3.46603500
H	-8.74237500	-0.71693700	-1.84923500
H	-9.02838700	1.00731900	-2.03796400
H	-2.85235300	-1.34937900	-0.25058200
C	-1.52377200	-5.52471900	-1.98505500
C	-1.01130100	-6.85140600	-2.35233700
C	-0.05204300	-9.41393800	-3.06505300
C	-1.87125700	-7.82212000	-2.88498100
C	0.33773900	-7.19107800	-2.17708600
C	0.80533200	-8.44436800	-2.53697300
C	-1.39881100	-9.07987000	-3.22544400
H	-2.92138000	-7.59048500	-3.00579800
H	1.02634600	-6.45858300	-1.77740500
H	1.85528000	-8.67665000	-2.40517000
H	-2.08880200	-9.81787400	-3.61650200
C	-2.32101200	-4.77642100	-2.83394200
C	-3.74486600	-3.02297800	-4.58712300
C	-2.35998800	-5.00347900	-4.24812000
C	-3.00230300	-4.15165600	-5.08566000
C	-3.73435600	-2.75668000	-3.25543400
H	-1.79701000	-5.83278900	-4.65210900

H	-2.96447700	-4.31594900	-6.15522200
H	-4.30529700	-1.92362400	-2.86873100
C	-4.51618900	-2.18556200	-5.56047900
H	-5.20361500	-2.80397600	-6.14264100
H	-5.08295900	-1.40404700	-5.05931700
H	-3.83227600	-1.71541900	-6.27313300
C	0.46595600	-10.76553700	-3.47080100
H	1.30812500	-11.07221200	-2.84949600
H	-0.31144000	-11.52697600	-3.40272100
H	0.81521600	-10.74522100	-4.50725600
C	-2.96686500	-3.55273500	-2.32105500
H	-3.43867500	-3.68954500	-1.35229300

Table S9. DFT coordinates of intermediate V-a

E= -2815.45996645 Hartrees

Sum of electronic and thermal Enthalpies= -2814.801632 Hartrees

C	0.83386700	1.29791600	-1.01903800
C	-0.05929000	1.54400400	0.09772600
C	-0.35661500	0.45508500	0.80885000
C	0.22468500	-0.83358800	0.32656700
S	1.29866600	-0.29982700	-1.22019700
C	1.04996700	2.51333800	-1.67845800
C	0.28780900	3.52335600	-1.07531100
S	1.96442300	3.09901100	-3.01801300
C	0.44842300	4.76259900	-1.69926600
C	1.32695200	4.67121500	-2.75934600
H	-0.03778500	5.68070700	-1.40873900
H	1.63554500	5.47469600	-3.41101000
C	1.07189200	-1.56391600	1.29564800
C	1.31651400	-2.92047000	1.17851300
S	2.00099000	-0.84228100	2.55231500
C	2.24532300	-3.36984500	2.16746000
C	2.69236400	-2.36371200	2.96884600
H	2.55797400	-4.39760400	2.27144300
H	3.37405900	-2.43257400	3.80134900
C	-0.52529500	3.00047600	0.10190900
C	-2.03755400	2.97257900	-0.18659300
C	-2.52708400	2.96446900	-1.49026400
C	-2.94157100	2.80416700	0.86257300
C	-3.88515900	2.79620200	-1.73794400
H	-1.85277400	3.07553300	-2.32904900

C	-4.29521200	2.63450500	0.60955200
H	-2.58747600	2.80351100	1.88515100
C	-4.79444600	2.62692100	-0.69545900
H	-4.23853500	2.79150000	-2.76218800
H	-4.97607900	2.50183300	1.44203700
C	-0.15427100	3.79160100	1.35977500
C	-0.71780300	5.05291200	1.56402100
C	0.79054600	3.33450800	2.27384500
C	-0.35284400	5.82479600	2.65625000
H	-1.45661200	5.43039300	0.86894500
C	1.15143100	4.11181800	3.36986400
H	1.25805400	2.36841800	2.14190200
C	0.58659400	5.36748300	3.58440400
H	-0.80656800	6.79939900	2.79208900
H	1.88647000	3.72955400	4.06810600
C	0.95048100	6.19555800	4.78678400
H	0.98489300	7.25779700	4.54007100
H	0.20808000	6.07065200	5.58002300
H	1.91924400	5.90436100	5.19336700
C	-6.26662300	2.46839800	-0.96209000
H	-6.44850300	2.05115800	-1.95296200
H	-6.73633600	1.81865800	-0.22246000
H	-6.77375800	3.43615300	-0.91454500
H	-1.01825000	0.43569800	1.66441500
C	0.72276700	-3.70033800	0.10376300
C	1.26625900	-5.05434400	-0.16399100
C	2.31974900	-7.63493100	-0.64316800
C	0.45350400	-6.18809400	-0.08748000
C	2.61662500	-5.23288500	-0.47825500
C	3.12993000	-6.49976400	-0.71963700
C	0.97319300	-7.45443900	-0.32344600
H	-0.59367800	-6.07414700	0.16319300
H	3.26685700	-4.36924900	-0.54386700
H	4.17760400	-6.60876900	-0.97468300
H	0.32061700	-8.31702000	-0.25535500
C	-0.29630800	-3.14411300	-0.61974900
C	-2.31408900	-1.81084000	-2.21518900
C	-0.87053900	-3.75455400	-1.79080900
C	-1.81206100	-3.13944900	-2.53684800
C	-1.84644000	-1.17863000	-1.13090500
H	-0.49137300	-4.71902300	-2.09814700
H	-2.19072600	-3.62622700	-3.42754900
H	-2.20838100	-0.18809000	-0.88448200
C	-3.32727400	-1.19134100	-3.13387100

H	-4.22992500	-1.80646400	-3.18227100
H	-3.60826400	-0.19089200	-2.80530600
H	-2.93425700	-1.12559000	-4.15214500
C	2.88827200	-9.01052200	-0.86642800
H	2.13445100	-9.69410600	-1.25887800
H	3.72726200	-8.98605100	-1.56298000
H	3.25585500	-9.43356900	0.07292600
C	-0.87912300	-1.80972300	-0.17277700
H	-1.43248200	-2.01748700	0.76068700

Table S10. DFT coordinates of compound 3

E= -2815.08422213 Hartrees
Sum of electronic and thermal Enthalpies= -2814.437184 Hartrees

S	10.85418800	12.37211600	7.23627300
S	8.81395100	10.04470700	5.37435300
S	9.98899900	8.81411600	1.87011400
C	12.80069500	9.19133500	5.91031400
C	13.90300200	9.42611400	4.86934800
C	15.23404200	9.52236500	5.28621000
H	15.48267400	9.35865400	6.32689500
C	16.24786700	9.81344700	4.38599700
H	17.26996500	9.87891700	4.74113300
C	15.97482400	10.02380900	3.03183900
C	14.64618500	9.94069200	2.62188700
H	14.39648400	10.10612100	1.58025200
C	13.62652500	9.64889900	3.52334400
H	12.60941700	9.60056200	3.16257200
C	17.08353800	10.30389200	2.05298900
H	16.70607200	10.78475800	1.15003500
H	17.57791300	9.37625800	1.75065300
C	12.51815300	10.50351300	6.60822800
C	13.26760200	11.41650500	7.37647100
H	14.31344900	11.31762600	7.62458700
C	12.49010400	12.47419500	7.77802900
H	12.79933600	13.32207000	8.36961900
C	11.20421600	10.88073700	6.44652900
C	10.43226200	9.94161700	5.68859900
C	11.39741300	8.88038800	5.30652300
C	13.12510500	8.06194600	6.90352100
C	13.85290700	6.93716900	6.51235500
H	14.30216900	6.90060900	5.52901700
C	14.01225000	5.85515600	7.36869000

H	14.58162700	4.99570600	7.03392900
C	13.45284600	5.85314100	8.64724100
C	12.73791600	6.98407700	9.04020100
H	12.29552400	7.01988400	10.02888800
C	12.57509200	8.06786700	8.18514600
H	11.99939100	8.91993400	8.52244300
C	13.59202800	4.65950800	9.55344700
H	12.84734100	3.89763600	9.30533800
C	11.16201400	7.73301300	4.66273900
H	12.00723900	7.05627100	4.56528000
C	9.89310100	7.19452100	4.14437200
C	9.23886600	6.16560200	4.86882000
C	9.75550800	5.71291800	6.11387400
H	10.64956000	6.18107100	6.50710500
C	9.15582900	4.71281900	6.83400500
C	7.97790900	4.11841700	6.30860400
H	7.49936700	3.31920200	6.86296500
C	7.43884200	4.53475800	5.12354700
H	6.54158800	4.06073000	4.75104400
C	8.03306400	5.57851000	4.35827800
C	7.48550200	6.02350400	3.12645400
C	8.15468500	7.01666300	2.41706200
C	9.35701300	7.58132900	2.93671000
C	7.78961300	7.63081800	1.16447700
H	6.90742200	7.35962300	0.60456700
C	8.66295600	8.57950300	0.76386600
H	8.61309700	9.17792900	-0.13246700
C	6.22215600	5.45618900	2.58644800
C	6.21918600	4.70604500	1.40891200
H	7.15449800	4.52201700	0.89472500
C	5.03603200	4.18730400	0.89637300
H	5.06378900	3.60116300	-0.01503700
C	3.81526100	4.40367800	1.53706800
C	3.82132600	5.15373900	2.71484400
H	2.88823100	5.33686100	3.23525200
C	5.00197200	5.67101200	3.23206900
H	4.98100800	6.24983500	4.14712300
C	2.52916700	3.87139700	0.96278000
H	2.06063600	4.61252700	0.30872200
H	1.81153300	3.63186200	1.74879600
H	2.70011200	2.97296900	0.36836600
H	13.44588600	4.93277900	10.59888700
H	14.57475000	4.19614900	9.45311000
H	17.84679400	10.94861100	2.49211400

C	9.71773300	4.24021100	8.14622800
H	10.59500800	4.81934700	8.43378100
H	8.97476900	4.32508800	8.94347500
H	10.00679900	3.18710800	8.09081600

Table S11. DFT coordinates of intermediate IV-b

E= -2815.49352677 Hartrees
Sum of electronic and thermal Enthalpies= -2814.835071 Hartrees

C	1.28199100	-1.29285300	-1.94371100
C	1.05073200	-0.22814800	-1.08301600
C	0.78127300	-0.63353100	0.22444200
C	0.82209100	-2.01265900	0.37055700
S	1.19691400	-2.80515900	-1.14998900
C	1.52432800	-0.78937100	-3.26278000
C	1.44752600	0.59120600	-3.23777100
S	1.89866600	-1.38888500	-4.82667100
C	1.70024900	1.17537700	-4.49636700
C	1.95306500	0.22456200	-5.45196200
H	1.69830700	2.23541800	-4.70198700
H	2.17041700	0.37917200	-6.49677700
C	0.57300300	-2.76063900	1.56655500
C	-0.32157200	-2.43925500	2.61586500
S	1.53831300	-4.12258400	1.99421400
C	-0.12858400	-3.29797800	3.75688100
C	0.78729100	-4.26932400	3.54600300
H	-0.72775100	-3.22664800	4.65143500
H	1.06109300	-5.08023000	4.20138800
C	1.16884100	1.10423300	-1.82317900
C	-0.13828500	1.89400500	-1.68535900
C	-1.09977800	1.92591100	-2.68937900
C	-0.39803700	2.59458600	-0.50408700
C	-2.27515900	2.65597700	-2.52846400
H	-0.93617600	1.38845700	-3.61358300
C	-1.57007800	3.31647500	-0.34576200
H	0.33593700	2.59331600	0.29187300
C	-2.53060200	3.36843100	-1.36048000
H	-3.00277600	2.66750100	-3.33132400
H	-1.74132500	3.85551500	0.57904000
C	2.38481100	1.93539100	-1.37834000
C	2.51275200	3.25473200	-1.82129700
C	3.41225200	1.40136800	-0.60698900

C	3.62803300	4.01095700	-1.49647400
H	1.72819400	3.69735100	-2.42165600
C	4.53076100	2.16507000	-0.28172100
H	3.35256100	0.38086500	-0.25401300
C	4.66027800	3.48176400	-0.71592800
H	3.69978400	5.03147300	-1.85488900
H	5.31423300	1.72202300	0.32203000
C	5.85433400	4.32056900	-0.34632200
H	5.59031300	5.05991300	0.41485800
H	6.66400000	3.70876700	0.05197900
H	6.23363800	4.86988500	-1.21018500
C	-3.80669100	4.14428100	-1.17256100
H	-4.37024600	4.21719500	-2.10266300
H	-4.44982900	3.66414100	-0.42930300
H	-3.60550300	5.15597900	-0.81478100
C	-1.39249000	-1.49630100	2.53898000
C	-2.12964400	-1.31106400	1.30703500
C	-3.47057900	-0.93100200	-1.14623100
C	-2.31742200	-2.38193900	0.40967700
C	-2.66075000	-0.05458400	0.95859300
C	-3.30976900	0.12557000	-0.24549800
C	-2.97814700	-2.19264700	-0.78497600
H	-1.95751700	-3.36616600	0.67461800
H	-2.50128900	0.79338700	1.60939300
H	-3.67124000	1.10802100	-0.51096900
H	-3.12062500	-3.03214500	-1.45375500
C	-1.78364600	-0.77847600	3.73071900
C	-2.51979400	0.64604700	6.05497000
C	-0.81722600	-0.39449800	4.68445000
C	-3.12909500	-0.43139700	3.97371900
C	-3.48364100	0.25491000	5.11751900
C	-1.18035400	0.31436600	5.81053800
H	0.22476300	-0.62132500	4.50520600
H	-3.89406700	-0.74243700	3.27649900
H	-4.52514800	0.49160700	5.29497000
H	-0.41807700	0.62584600	6.51345200
C	-2.91331100	1.37501100	7.30415300
H	-3.83044200	1.94625900	7.16203600
H	-3.09390300	0.65893400	8.11167800
H	-2.12378800	2.04842800	7.63799600
C	-4.11677300	-0.70823600	-2.47977700
H	-3.35382800	-0.43963500	-3.21680100
H	-4.61875700	-1.60701700	-2.83815800
H	-4.83496300	0.11080800	-2.44439500

H	0.58595100	0.02980500	1.05312500

Table S12. DFT coordinates of TS-b

E= -2815.45022154 Hartrees
Sum of electronic and thermal Enthalpies= -2814.792805 Hartrees

C	-0.87325500	-1.92014700	-4.21639200
C	-1.17042000	-1.06039400	-3.20050300
C	-1.28603900	-1.72077600	-1.91633800
C	-1.01845900	-3.15057800	-2.09801800
S	-0.70509100	-3.58953800	-3.71021600
C	-0.59220200	-1.19649100	-5.42715300
C	-0.68184100	0.15132200	-5.15212800
S	-0.15890100	-1.50169000	-7.05753100
C	-0.39436900	0.95892600	-6.27322800
C	-0.10012800	0.19815600	-7.37500300
H	-0.39846200	2.03862800	-6.28058500
H	0.14974000	0.54039500	-8.36661600
C	-1.36924900	-3.90815500	-0.97322400
C	-2.27552500	-3.20267600	-0.17693200
S	-0.95651600	-5.43133700	-0.28679600
C	-2.56798900	-3.86437200	1.03012000
C	-1.93513900	-5.08013400	1.09151900
H	-3.23955300	-3.49811100	1.79113200
H	-2.00434600	-5.81154300	1.88182800
C	-0.98689300	0.39106900	-3.67281700
C	-2.24333400	1.22461400	-3.41811700
C	-3.20513400	1.42130000	-4.40503100
C	-2.48894000	1.74345500	-2.14461800
C	-4.38619800	2.10097600	-4.12213500
H	-3.05030700	1.02520400	-5.39917200
C	-3.66338200	2.42501400	-1.86927200
H	-1.76185000	1.60717300	-1.35558500
C	-4.63997900	2.61132100	-2.85085200
H	-5.12281800	2.22842200	-4.90650300
H	-3.83228100	2.80160000	-0.86832700
C	0.27847400	1.03414700	-3.06189600
C	0.44167000	2.42003800	-3.11616700

C	1.32605000	0.27435400	-2.54558800
C	1.60129500	3.02140300	-2.64885400
H	-0.34601500	3.03926300	-3.52400100
C	2.48631100	0.88165200	-2.07609700
H	1.26184000	-0.80467800	-2.51817700
C	2.64521500	2.26548100	-2.11110100
H	1.69667900	4.09962200	-2.70468700
H	3.28148400	0.26194600	-1.67846800
C	3.88357500	2.92737800	-1.56973900
H	4.15986400	3.79868800	-2.16546700
H	3.72134500	3.27336900	-0.54494000
H	4.72920900	2.23916700	-1.55434500
C	-5.91359600	3.34689200	-2.53364700
H	-6.64529200	3.24355500	-3.33486700
H	-6.36119700	2.97316200	-1.61031700
H	-5.72268300	4.41361700	-2.39008800
C	-2.84886500	-2.00028600	-0.84030900
C	-3.92647500	-2.32467400	-1.84304200
C	-5.92595400	-2.90438600	-3.76049900
C	-4.45738900	-3.61089800	-1.96221700
C	-4.42025400	-1.33220300	-2.69536200
C	-5.39586800	-1.61658800	-3.63345400
C	-5.43998200	-3.89104800	-2.90555600
H	-4.12084900	-4.40604000	-1.31201300
H	-4.03721200	-0.32815600	-2.62246700
H	-5.74700900	-0.82329800	-4.28248600
H	-5.83405400	-4.89803200	-2.97103100
C	-3.09459900	-0.85168100	0.08425800
C	-3.52794400	1.25281200	1.92645700
C	-2.06218400	-0.38410500	0.90874600
C	-4.35608500	-0.26711600	0.22767300
C	-4.56548700	0.75932600	1.13695300
C	-2.27107500	0.65099900	1.80201000
H	-1.07539100	-0.82809900	0.85133300
H	-5.18734300	-0.62077700	-0.36309500
H	-5.55541300	1.19003800	1.22572100
H	-1.44789200	1.00077700	2.41289000
C	-3.74207100	2.41212200	2.85767400
H	-3.52250200	3.35434400	2.34690500
H	-4.77511300	2.46054200	3.20285200
H	-3.08617900	2.35239800	3.72673600
C	-6.99955300	-3.19738100	-4.77165300
H	-7.95780300	-2.78205500	-4.44790200
H	-6.76373200	-2.74647900	-5.73737000

H	-7.13267900	-4.26951000	-4.91520400
H	-0.71863600	-1.26526100	-1.11439500

Table S13. DFT coordinates of intermediate V-b

E= -2815.45399658 Hartrees
Sum of electronic and thermal Enthalpies= -2814.794796 Hartrees

C	1.13260200	-1.11118500	-1.93303800
C	0.69910600	-0.32395500	-0.91726800
C	0.61443500	-1.04355600	0.37466500
C	0.99695000	-2.47251000	0.10940800
S	1.43961300	-2.79130700	-1.48425700
C	1.45267700	-0.32493800	-3.09845900
C	1.25846700	1.00022500	-2.78247100
S	1.99202100	-0.53851300	-4.71101700
C	1.54898200	1.86926400	-3.85679700
C	1.95458600	1.17310800	-4.96524000
H	1.46999200	2.94572300	-3.82628000
H	2.23824300	1.56851200	-5.92751200
C	0.69634000	-3.24239700	1.22877500
C	-0.17269300	-2.51744100	2.05720300
S	1.06313700	-4.79691200	1.87847400
C	-0.49283200	-3.19899300	3.23442300
C	0.10925200	-4.43916200	3.26353300
H	-1.14587900	-2.83208300	4.01110100
H	0.02902400	-5.17860800	4.04590400
C	0.85762400	1.15925500	-1.31542900
C	-0.42724200	1.95909900	-1.10224100
C	-1.36739700	2.09357100	-2.12105600
C	-0.73391300	2.49545700	0.15033500
C	-2.58130400	2.73174600	-1.89099000
H	-1.16729300	1.67954100	-3.09991000
C	-1.94448000	3.13308800	0.37501700
H	-0.03307300	2.40075500	0.96712400
C	-2.89523400	3.26053900	-0.64017200
H	-3.29824100	2.81013300	-2.69963100
H	-2.15974100	3.52016500	1.36350500
C	2.08740900	1.79102300	-0.61473400

C	2.20627900	3.18006100	-0.53667400
C	3.15907000	1.02345900	-0.15969300
C	3.33595400	3.77226000	0.01065800
H	1.40969700	3.81145700	-0.90475300
C	4.28774800	1.62028700	0.39068400
H	3.14582000	-0.05375800	-0.25299800
C	4.39592000	3.00547300	0.49718500
H	3.39346800	4.85355700	0.05737500
H	5.09961100	0.99165900	0.73707100
C	5.59900300	3.65232800	1.12825200
H	5.41115700	3.87271600	2.18295200
H	6.47289600	3.00196300	1.07933000
H	5.84439100	4.59595600	0.63881400
C	-4.20366100	3.95898200	-0.38746500
H	-4.93319700	3.73346000	-1.16551200
H	-4.62624800	3.66636900	0.57545100
H	-4.06702100	5.04357700	-0.36438700
C	-0.65904600	-1.25405300	1.38359700
C	-1.89813400	-1.57358600	0.52417200
C	-4.13214300	-2.10243800	-1.13953700
C	-2.44318600	-2.85346200	0.43484600
C	-2.50139600	-0.56012700	-0.22272100
C	-3.59132400	-0.81827200	-1.03672000
C	-3.54027900	-3.10989700	-0.38378300
H	-2.03248300	-3.67474900	1.00395900
H	-2.13031000	0.44733500	-0.15273800
H	-4.02822800	-0.00208400	-1.59962700
H	-3.93882200	-4.11649700	-0.42607500
C	-0.85311200	-0.11638700	2.37728400
C	-1.18371300	1.96763600	4.26815100
C	0.24761200	0.47660200	3.00292900
C	-2.12252400	0.32239400	2.75139400
C	-2.28191600	1.34443400	3.67870100
C	0.08656600	1.50125500	3.92226200
H	1.25520300	0.15229800	2.77484400
H	-3.00160200	-0.12644900	2.31318400
H	-3.28283600	1.66727700	3.93914500
H	0.96307900	1.94949200	4.37440900
C	-1.35465700	3.11946500	5.21948200
H	-2.32183700	3.08170800	5.72146200
H	-0.57046500	3.12922000	5.97755100
H	-1.30025900	4.07064300	4.68219200
C	-5.30384500	-2.37539700	-2.04265000
H	-5.00409500	-2.32061200	-3.09261500

H	-5.72292600	-3.36593000	-1.86611600
H	-6.09446100	-1.63705700	-1.89537700
H	1.39559400	-0.63394400	1.02148400

Table S14. DFT coordinates of compound 2

E= -2815.05803149 Hartrees

Sum of electronic and thermal Enthalpies=-2814.411166 Hartrees

C	1.81230300	-0.80453500	-2.05787600
C	0.95251500	-0.26345800	-1.10975400
C	0.47671000	-1.25440900	-0.21888000
C	1.01426300	-2.50155500	-0.51328800
S	2.07378800	-2.49809100	-1.87296200
C	2.25732500	0.22060600	-2.96044700
C	1.68158400	1.41694000	-2.59913400
S	3.27348300	0.38537500	-4.33533500
C	2.05896200	2.48931100	-3.43742400
C	2.91589300	2.08226000	-4.42582200
H	1.72294800	3.51012900	-3.32903300
H	3.36282500	2.67736500	-5.20581300
C	0.53077900	-3.47892700	0.42213300
C	-0.31049600	-2.86269200	1.31970600
S	0.69579300	-5.15806500	0.74445300
C	-0.83587600	-3.74898700	2.28592400
C	-0.37715900	-5.02693700	2.10344700
H	-1.51544300	-3.47172100	3.07840400
H	-0.60468900	-5.90517500	2.68588000
C	0.80347700	1.25321800	-1.35264900
C	-0.66452300	1.59569500	-1.65032400
C	-1.21189300	1.26922000	-2.89379800
C	-1.50798000	2.16558400	-0.70248200
C	-2.55155800	1.49688800	-3.16895700
H	-0.58248500	0.82509300	-3.65446800
C	-2.85149900	2.39467400	-0.98062900
H	-1.11756600	2.44178500	0.26475300
C	-3.40155400	2.05945800	-2.21389400
H	-2.94660600	1.22721100	-4.14195700
H	-3.48158500	2.83199400	-0.21480200
C	1.45389900	2.10774800	-0.25462200
C	1.27238900	3.49369400	-0.24048200
C	2.33304100	1.56017200	0.67441700
C	1.92175800	4.29141500	0.69034100
H	0.61111200	3.95643900	-0.96117800
C	2.98946100	2.36290900	1.59985300
H	2.50157400	0.49354600	0.69391000
C	2.79094000	3.74029900	1.63487000
H	1.75348300	5.36247800	0.67994800

H	3.65337900	1.89922200	2.31938800
C	3.45623200	4.60426700	2.67270800
H	2.77030300	4.81840600	3.49742400
H	4.33450900	4.11480100	3.09531600
H	3.76688000	5.56309100	2.25370200
C	-4.86600300	2.24988500	-2.50070300
H	-5.39633200	1.29595700	-2.43241300
H	-5.32778900	2.93435000	-1.78848400
H	-5.02906000	2.63983200	-3.50716600
C	-0.46785900	-1.37222200	0.99586600
C	-1.94632000	-1.16654500	0.63430400
C	-4.70269000	-0.98602600	0.00689000
C	-2.91247600	-1.08074700	1.64095500
C	-2.38616800	-1.18525500	-0.68548600
C	-3.73919100	-1.09802600	-0.99157600
C	-4.26152600	-0.98505900	1.33232400
H	-2.60821400	-1.08287400	2.67933600
H	-1.67050400	-1.25303400	-1.49153800
H	-4.04323900	-1.09772100	-2.03133100
H	-4.98536400	-0.91202900	2.13628300
C	0.04227200	-0.48984800	2.14564800
C	1.12609100	1.15657900	4.17996500
C	1.15391700	-0.89584500	2.88829600
C	-0.51898600	0.74696200	2.44592100
C	0.01266900	1.55405600	3.44615900
C	1.68361200	-0.08941500	3.88414300
H	1.61441500	-1.85336700	2.68071200
H	-1.38498200	1.08869100	1.90041000
H	-0.44501300	2.51579800	3.64699200
H	2.54990400	-0.43156100	4.43907600
C	1.74054200	2.04939600	5.22327000
H	1.04655300	2.82696600	5.54391100
H	2.05113100	1.48202000	6.10252100
H	2.63038400	2.54574900	4.82616100
C	-6.16354000	-0.83417300	-0.32318800
H	-6.38169100	-1.17918200	-1.33452200
H	-6.78987500	-1.39546000	0.37265100
H	-6.46896200	0.21425000	-0.26013100

Table S15. DFT coordinates of water

E= -76.46933837 Hartrees
Sum of electronic and thermal Enthalpies= -76.444452 Hartrees

O	0.68032400	0.17738800	0.00000000
H	1.64342900	0.21781400	0.00000000
H	0.39694400	1.09874000	0.00000000

References

1. G. M. Sheldrick, *Acta Crystallogr., Sect. C: Struct. Chem.* 2015, **C71**, 3-8.
2. DIAMOND – Visual Crystal Structure Information System, CRYSTAL IMPACT, Postfach 1251, 53002 Bonn, Germany, 2001.
